# Novel delivery strategy: finasteride-loaded solid lipid nanoparticles for improved androgenetic alopecia therapy†

**DOI:** 10.1039/d5ra00399g

**Published:** 2025-06-04

**Authors:** Harekrishna Roy, Balaji Maddiboyina, Bhabani Shankar Nayak, Raghvendra A. Bohara

**Affiliations:** a Department of Pharmaceutics, Nirmala College of Pharmacy Mangalagiri Guntur Andhra Pradesh India hareroy@gmail.com; b Scientific Writing Services, Medical and Scientific Communications CoE, Freyr Global Regulatory Solutions & Services, Phoenix SEZ Hyderabad India; c KIIT School of Pharmacy, KIMS, KIIT Deemed to be University Bhubaneswar Odisha India; d D Y Patil Education Society Kolhapur MS India raghvendrabohara@gmail.com

## Abstract

Androgenetic alopecia (AGA) is currently the most prevalent cause of hair loss on the scalp. The daily administration of finasteride (FINA) by oral route may lead to the development of numerous undesirable systemic side effects. However, commercially available dermal dosage forms are available only with minoxidil; few studies have claimed severe side effects. Our study deals with the development of solid lipid nanoparticles (SLNs) of FINA with a suitable combination of l-α-phosphatidylcholine (LPC) and *N*-trimethyl chitosan (NTC) to overcome limitations along with good skin retention and hair growth. FINA-SLNs were developed using the ultrasonication technique and characterized further, along with hair growth observed in the animal model. The formulation NP7 showed the highest zeta potential value of −16.5 mV. The absence of the =NH peak in the ^1^H-NMR spectra could be due to the protons attached, which have substantial exchangeability and result in a probable disappearance in the NMR spectra. The investigation showed the highest skin retention of 226.76 μg of FINA by NP7, along with a modest amount of FINA permeated (71.23 μg) during the study period of 18 h. The animal model using C57BL/6 mice showed a notable enhancement in hair covering and growth in Group IV, which received treatment without any visible cutaneous reaction on the skin. This outcome underscores the effectiveness and importance of the formulation developed using a suitable combination of LPC and NTC, which could be used to manage AGA effectively.

## Abstract 2

Androgenetic alopecia (AGA) is currently the most prevalent cause of hair loss on the scalp.

## Graphical abstract

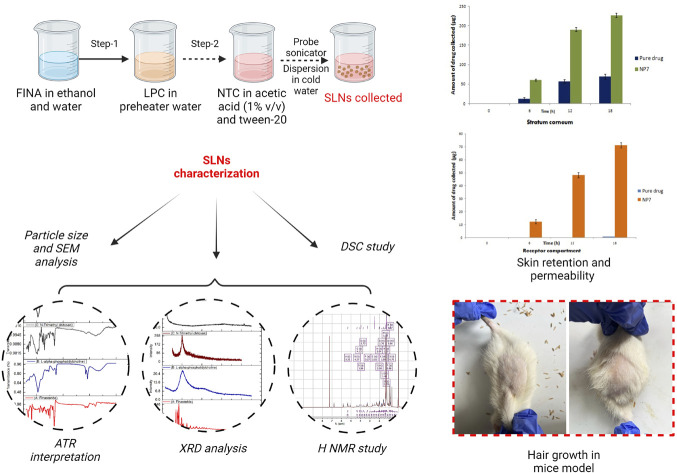

## 1 Introduction

Androgenetic alopecia (AGA) exhibits a noticeable hereditary tendency and is reasonably attributed to an exaggerated sensitivity to androgen.^1^ This condition is a genetically determined disorder by exaggerated reaction to androgens, impacting around 50% of both males and girls.^2,3^ There is a prevailing belief that the enzyme 5 alpha reductase converts testosterone into dihydrotestosterone (DHT). This compound exhibits a significantly higher affinity for the androgen receptor.^4^ Moreover, polymorphisms in the androgen receptor gene are linked to AGA. Studies shows genetic variability at the androgen receptor region is considered the primary factor influencing the initial phase of AGA.^5,6^

Lipid-based nanoparticles (LNPs) provide a range of advantages,^7^ including the protection of pharmaceuticals against degradation within the body, improvement of drug solubility, facilitation of permeability and drug absorption, facilitation of targeted drug delivery to specific disease sites, maintenance of controlled drug release, and modification of drug biodistribution.^8–11^

SLNs are special category of LNP are minute, spherical particles that possess a solid lipid core when maintained at ambient temperature.^12^ Within the realm of SLNs, the active substance can be integrated into the lipid core, lipid shell, or evenly distributed throughout the lipid matrix.^13,14^ There are several technological advantages associated with their use, such as protecting drugs from degradation, improving stability, increasing the encapsulation of hydrophobic drugs,^15^ facilitating large-scale production,^16^ eliminating toxic organic solvents,^17^ improving the bioavailability, and enabling the simultaneous adminstration of a large number of therapeutic molecules.^18,19^ In industrial scale, various methods for the preparation of SLNs including high-pressure homogenization, solvent emulsification–evaporation, and microemulsion techniques are widely being utilized for development. Those techniques are easy to use and do not require complex machinery to finalize the dosage form.

l-α-Phosphatidylcholine (LPC) is a phospholipid variant that can be serves as a primary constituent in nanostructure delivery systems, such as lipid nanoparticles, and also contributes to the stabilization of liposomal membranes.^20,21^ In a recent study conducted by Vasileva *et al.*,^22^ lipid nanoparticles containing Rotenone were successfully synthesized using the lipid film hydration method. The authors observed significant cytotoxicity against pancreatic cancer and demonstrated enhanced penetration through the use of confocal microscopy. Additionally, it was noted the significant increase in fluorescence intensity within the cells when exposed to formulation, indicated a high uptake by the specialized PANC-1 and HuTu 80 cells. Skóra B. *et al.*,^23^ conducted an independent study wherein they observed successful entrapment of silver nanoparticles in LPC. A decrease in caspase-3 activity was seen while using LPC-silver nanoparticles in comparison to silver nanoparticles. Qiu Y. *et al.*,^24^ did a study wherein they developed nanoparticles composed of phosphatidylcholine-chitosan loaded with gentamicin. The study demonstrated a significant permeability and antibacterial efficacy, as well as a notable macrophage engulf response while exhibiting minimal cytotoxicity. In a recent study conducted by Khan M. S. *et al.*,^25^ the researchers successfully developed hybrid nanoparticles having phosphatidylcholine-poly-lactic acid-*co*-glycolic acid loaded with triamcinolone acetonide. Those nanoparticles were specifically designed for the purpose of topical administration. The researchers reported a specific size of 163 nm with a high loading efficiency.

*N*-Trimethyl chitosan (NTC) is an excipient produced from chitosan that posses enhanced biocompatibility, bioadhesive characteristics, and increased solubility.^26,27^ According to numerous reports, it has been observed that NTCs offer the ability to enhance the bioavailability of drugs,^28^ facilitate vaccine delivery,^29^ enable the regulated release of drugs,^30^ and exhibit organ-specific targeting capabilities.^31^

Finasteride (FINA) has been recognized by the United States Food and Drug Administration (USFDA) as an approved androgen antagonist for the treatment of AGA. However commercially topically applied formulations of FINA available along in combination only with minoxidil (increase blood flow to scalp), documented for effective treatment which are approved by USFDA. Nevertheless, minoxidil can lead to significant adverse effects such as graying of hair, scalp burning, and headaches, as extensively documented in several research.^32,33^ Those combined drugs in a formulation are typically available in the form of gels or lotions. Meanwhile, in several study it documented that, the systemic administration of FINA (tablet dosage approved by USFDA) has been observed to result in significant adverse effects, such as diminished libido,^34^ erectile dysfunction,^35^ and male infertility.^36^

Numerous investigations have explored various topical novel formulations of FINA, including liposomes, Transethosomes, and microemulsions.^37,38^ In contrast, some of those formulations highlighted minimal skin retention of FINA, as well as always associated with several demerits including high cost of raw materials, stability issues and difficulty in prolonging drug release. Our study encompassed the development of SLNs by ultrasonication technique. There have been several reports published as mentioned earlier for SLN preparation. However, there are potential advantages of ultrasonication technique observed in SLNs preparation which is used in current research including uniform particle size, ensuring uniformity in particle size, simple and cost-effective and high encapsulation efficiency within the lipid matrix. In this investigation, we documented transdermal delivery of FINA-SLN produced in an appropriate combination of LPC and NTC, which is controlled by varying the amount of Phosphatidylcholine and NTC. It also attempted to give longer FINA release through skin while focusing on higher epidermal retention and hair growth in a C57BL/6 mouse model. According to the literature, we included LPC to improve SC retention and entrapment, whilst NTC would provide improved bioadhesive properties. The graphical abstract depicts the process of creating and characterizing the SLN.

## 2 Material and methods

### 2.1. Materials

FINA was procured from Yarrow chem. products, Mumbai, India. LPC was procured from Sigma-Aldrich, Mumbai, India. NTC was obtained from Loba Chemie, Ahemdabad, India. Ethanol and Acetic acid obtained from Krishna Pharma, Hyderabad, India. The solvents used were highly pure.

### 2.2. Method

**Fig. 1:**
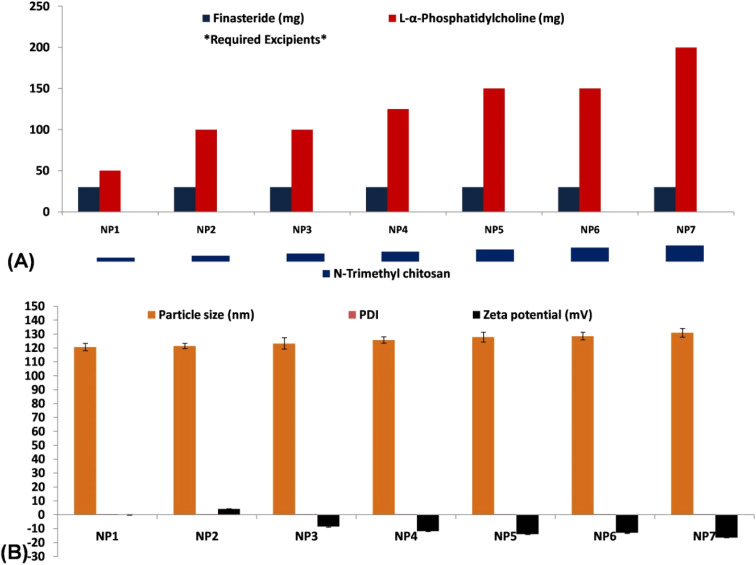
(A) The SLNs composition formulated using the ultrasonication technique denoted as NP1–NP7; FINA amount fixed at 30 mg and LPC fixed at a range of 50–200 mg; a 0.5 to 2% of NTC considered in SLN development; (B) particle size, PDI and zeta potential values of prepared SLNs. The value of PDI was determined to be ranged from 0.375 to 0.463. SLNs displayed a highest zeta potential of −16.5 mV and +4.2 mV as the least zeta value obtained from NP2.

The SLNs were formulated using the ultrasonication technique as per the procedure established by Kumar R. *et al.*,^39^ with minute modification and quantities incorporated highlighted in Fig. 1A (required amount can be found in ESI file†). In this experiment, LPC was dispersed in purified water that had been preheated to a temperature of 60 °C. In a separate beaker, a certain amount of the FINA was combined with a mixture of ethanol (60 parts) and water (40 parts). The FINA solution was poured into the lipid phase with constant agitation utilizing a ULTRA-TURRAX® T18 mixer (IKA, Germany), while ensuring the temperature mentioned earlier, in order to generate a lipid phase incorporating the drug. Furthermore, solution of NTC was thoroughly mixed with tween-20 (1%) in a solution of acetic acid (1% v/v). The lipid phase was ultimately disseminated into a solution of NTC, with the stirring speed maintained at 10 000 rpm for duration of 5 minutes, resulting in the formation of a microemulsion. In order to decrease the size of the globules, the microemulsion was subjected to sonication at room temperature using a probe sonicator (UAI-PS20khz-900W, Ultra Autosonic, India) for a period of 10 minutes at amplitude of 6%. The SLNs were collected at the bottom of container by dispersing in cold water (2–5 °C) for one hour, with stirring continuously at a 500 rpm utilizing a magnetic stirrer (Remi 2 MLH, Mumbai, India).

### 2.3. The entrapment efficiency (EE) and drug loading (DL)

The procedure conducted for determination of EE (eqn (1)) and DL (eqn (2)) was adopted from the method enumerated by Pi *et al.*,^40^ with slight modification. The experimental setup involved the utilization of a high-speed centrifugal filter (Remi R-8C, Hyderabad, India) for a time of 10 min. A volume of 5 ml of SLN dispersion was processed during this operation. The supernatant solution underwent measurement utilizing a UV-visible spectrophotometer at 255 nm (Double beam, Shimadzu, UV 3600i, Japan) after undergoing centrifugation. Similarly, the determination of the total amount of FINA in the sample (DL) was performed using a bath sonicator (Ultrasonicator, Verilux, Hong Kong) in a mixture of ethanol and water. A predetermined quantity (50 mg) of SLN was dispersed in a 50 ml volumetric flask containing ethanol and subjected to bath sonication for 10 min. Finally the supernatant liquid was collected and filtered by whatman filter paper (45 micron). The filtrate analyzed for FINA in the specific SLN formulation utilizing UV-visible spectrophotometer.1
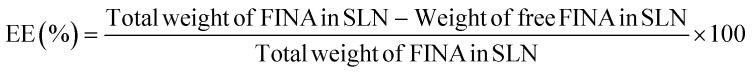
2
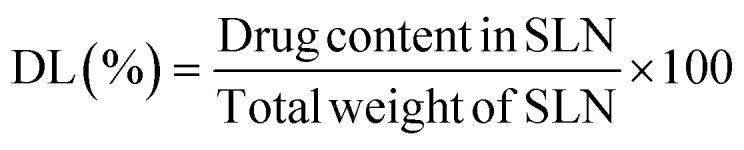


### 2.4. *In vitro* release performance of FINA-SLN

To evaluate the drug release of FINA from the formulated preparations, an *in vitro* method was processed, utilizing the diffusion technique with a Franz-diffusion cell, following the protocol described by Kaur R. *et al.*^41^ Before being used, the dialysis cellophane membrane layer was cut into segments of equal size, measuring 6 cm × 2.5 cm. A dispersion sample, with a volume of 2 ml (equivalent to 10 mg of the drug dissolved in deionized water), was introduced into the donor compartment. Aliquots of 0.5 ml were extracted at a predetermined time and then subsequently substituted with an equal volume of newly prepared buffer solution. The aliquots were diluted and detected by UV spectrophotometer.

### 2.5. Morphological study and size characterization

The scanning electron microscope (SEM) is frequently employed for examination of dispersed nanometer-sized dosage forms, enabling the assessment of their distribution and morphology. The SEM (HITACHI S-3700, Japan) was set to a working distance of 15 000 microns and an accelerating voltage of 14 500 volts, as reported by Maddiboyina B. *et al.*^42^ The process of SLNs size determination and polydispersity index (PDI) is carried out with the SZ-100V2 instrument, Horiba Scientific, India. In this study, the zeta potential, is measured by the Malvern apparatus, United Kingdom, utilized to quantify the electrical potential difference between the SLN and the dispersion medium.

### 2.6. ^1^H-NMR spectroscopy investigation

In the present investigation, the samples were prepared through the dispersion of 100 mg of the SLN in a solvent system. The Nuclear magnetic resonance (NMR) measurements were conducted at a temperature of 25 °C with a magnetic field strength of 500 MHz.^43,44^ All ^1^H-NMR experiments were performed using a Bruker BioSpin GmbH, Germany (500 MHz, DMSO and CDCl3; data interpretated by TopSpin^®^) at the Osmania University, Hyderabad.

### 2.7. Compatibility study by attenuated total reflectance (ATR)

The examination in this study was conducted using ATR spectroscopy, employing the ATR machine (Bruker-OPUS; V 7.5 instrument, Germany). Spectral analysis was performed on the FINA, its constituent formulations, and the selected SLN at 1.0 cm^−1^ resolution, including wave number from 4000 to 600 cm^−1^.^45^

### 2.8. Differential scanning colorimetry (DSC) observation

The DSC equipment utilized in this study was DSC 60A, Shimadzu Corporation, Japan. The thermograms were acquired and subsequently processed for further investigation as per the procedure narrated by Motas J. G. *et al.*^46^

### 2.9. XRD study

The use of X-ray diffraction (XRD) analysis lets us look closely at a crystalline substance, especially when the crystals have a preferred orientation. The experiment was performed using a Shimadzu X-ray diffractometer (model XRD-7000, manufactured in Japan) as per the methodology detailed by Whba F. *et al.*^47^ The diffractometer was calibrated properly, and the sample was placed in the sample holder while maintaining alignment on the X-ray beam path. Various measurement parameters such as scan, range and speed selected. The scan was turned on and diffractogram was obtained. The peak positions and intensities were further analyzed to interpret the information.

### 2.10. *In vitro* skin retention and permeability study

#### 2.10.1 *In vitro* skin retention and permeability study was performed on porcine skin obtained from Mangalagiri Local Market, Andhra Pradesh

We employed the Franz diffusion cell apparatus to quantify the transdermal permeability and mechanism of drug release from FINA-SLN. The approach was implemented using the methodology outlined by various previous researchers.^48–50^ The donor chamber was filled with 2 ml of chosen FINA-SLN formulation. The receptor chamber, on the other hand, was filled with buffer solution (phosphate buffer = pH of 6.8) rotated at a speed of 100 rpm. The preparation of the FINA-SLN gel involved the dispersion of SLN in hydroxypropyl methylcellulose (HPMC-15 CPS) at 2% w/v. Porcine ear skin (collected from local market) is included for dermal retention and permeation studies. The skin that had been prepared was positioned at the junction between the donor and receptor compartments. The receptor fluid was collected at particular time intervals and thereafter subjected to analysis. The stratum corneum (SC) was stripped through the utilization of the tape-stripping technique. The skin sample underwent a process of 70 iterations of stripping in order to fully eliminate the SC layer from the skin. The tape was introduced into a volumetric flask containing 10 ml of ethanol. The flask underwent sonication for duration of 20 minutes, followed by centrifugation at a speed of 10 000 rpm for a period of 10 minutes. The aliquots were subjected to analysis using High-Performance Liquid Chromatography (HPLC, Nexera lite, Shimadzu, Japan).

### 2.11. Effect of SLN in hair growth

#### 2.11.1 All animal procedures were performed following the guidelines for Care and Use of Laboratory Animals, approved by the Ethics Committee of Nirmala College of Pharmacy, Mangalagiri, Affiliated to ANU University, SBTET (AP). The work received approval from the institutional animal ethical committee under the reference number NCP/PhD/23-24/036

The methodology employed in the study conducted by Palakkal *et al.*,^51^ was implemented with minor adjustments. Male C57BL/6 mice aged 6 months were selected for the study. In the present investigation, subcutaneous administration of DHT injection at a dosage of 1 mg kg^−1^, with a volume of 100 μl, was employed as a means to cause alopecia.^52^ Group I was designated as the model group, receiving no treatment. Group II, the positive control group, was treated with a 5% marketed gel (FIN-XL; Minoxidil and FINA) applied topically daily. Group III received FINA in HPMC-15 CPS gel daily. Group IV received NP7, an equivalent dose of 1 mg kg^−1^ of FINA, also daily. The group of mice was administered daily injections of DHT. The duration of the process spanned 28 days.^53^ The dorsal skin was carefully trimmed (3.0 cm^2^ area) using a trimmer to facilitate the clear observation of hair growth.

## 3 Results and discussion

### 3.1. FINA-SLN release study and *in vitro* analysis

**Fig. 2:**
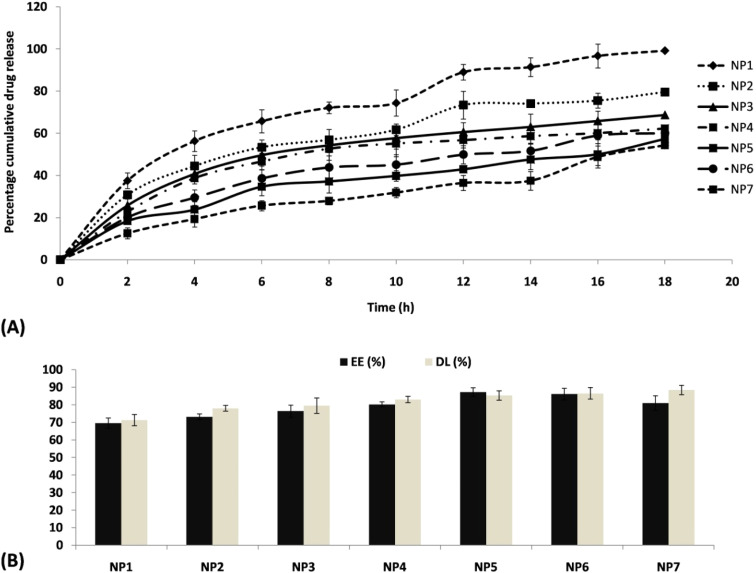
(A) *In vitro* percentage of FINA release from SLNs, an *in vitro* method was processed, utilizing the diffusion technique with a Franz-diffusion cell (NP1–NP7). It exhibited a notable abrupt reduction in its release pattern, potentially because to the predominant presence of a substantial quantity (200 mg) of LPC and 2.0% of NTC; (B) EE and DL of developed formulations. The investigation determined that the range of the DL was within the range of 71.36% to 88.52%, indicating a satisfactory level.

The assessment of the impact of drug and excipient combinations is a pivotal component in the field of drug release investigation. The data obtained from the study underwent analysis and subsequently was reported in Fig. 2A. All formulations demonstrated a release rate of 10% during the initial 2 h period of diffusion testing. Based on the data provided, it is evident that the formulation denoted as “NP7” had the lowest and steadiest drug release rate, recording a value of 54.25% over a period of 18 h. In contrast, the formulation referred to as “NP1” demonstrated the highest amount of drug release, with a value of 99.13% in the previously given timeframe. The data on the release of the drug was recorded and analyzed, revealing a progressive decrease in the percentage of drug release as the quantity of LPC increased. The sustained release of the drug was seen in the SLNs, likely attributed to the inclusion of LPC. This formulation facilitated the dispersion of FINA throughout the nanoparticle, hence impeding the diffusion of the drug into the release medium. In a similar study, the extended release of rosuvastatin from flexible lipid-based nanoparticles produced by LPC was documented and investigated by Amhed T. A. *et al.*^54^ They observed a comparable trend of drug release in conjunction with the progressive augmentation of NTC. Similarly, a research conducted by Roy H. *et al.*,^55^ provides support for the role of NTC, as they investigated the use of NTC to load and control the release of tofacitinib citrate SLN. In our current study, an equal amount of LPC (150 mg) was incorporated into both NP5 and NP6. However, NP5 contained 1.5% NTC, whereas NP6 contained 1.75%. It is noteworthy that NP6 demonstrated a larger percentage of FINA released (60.08%) in comparison to NP5 (57.24%) during a time frame of 18 h. It may be ascertained that a significant concentration of NTC (1.75%) has the potential to create a complex with FINA that is soluble in water, resulting in increased release efficacy. This conclusion is consistent with the findings reported by Ali Y. A. *et al.*,^56^ who synthesized zinc oxide nanoparticles and stabilized them using water-soluble NTC. Similarly in another study, Li S. *et al.*,^57^ conducted a study wherein they observed an increase in the bioavailability and antioxidant activity of vitexin when it was coated with NTC. Nevertheless, NP7 exhibited a notable abrupt reduction in its release pattern, potentially because to the predominant presence of a substantial quantity (200 mg) of LPC and 2.0% of NTC; selected as promising formulation for further study. The precise mechanism responsible for the release of pharmaceuticals from created SLNs has been determined, indicating that the formulations exhibit the Higuchi pattern of drug release. This observation serves as empirical evidence supporting the hypothesis that the drug is evenly distributed throughout the nanoparticulate dose form.

### 3.2. EE and DL characterization of SLNs

The determination of DL and EE in a pharmaceutical formulation has significant variation. It is influenced by several parameters, including the polarity of the vehicle, the selection of polymer, the quantity used, and interactions involved. The data obtained are included in Fig. 2B. The evidence presented in this statement is further supported by the research conducted by Elkateb *et al.*,^58^ whereby they showed a significant increase in DL in the nanostructure lipid dosage form of darunavir and ritonavir. Formulation “NP7” exhibited highest DL of 88.52% and the least by “NP1” with 71.36%. This could be the presence of higher amount of NTC (2%) which made a layer surrounding the SLN and retained greater amount of FINA. It noted a gradual increase in DL with a progressive increment of NTC and LPC. Similarly, the determination of EE was carried out using an appropriate methodology, as described earlier. The recorded data exhibited a range from 69.58% to 87.25% of entrapment. Among the several formulations examined, it was observed that “NP1” displayed the lowest drug concentration, whereas “NP5” exhibited the maximum EE. The findings of the study indicated that an elevated concentration of LPC led to enhanced EE. The study's findings demonstrated that the incorporation of lipids resulted in an elevation of EE and a decrease in drug leakage from the polymeric core.^59,60^ It noted an increase in lipid quantity from 50 to 150 mg resulting in an improvement in entrapment efficiency. However, a subsequent increase in the quantity from 150 to 200 mg led to a steady loss in EE, as observed in NP6 and NP7. The deterioration of SLN morphology was attributed to the augmentation of lipid content. The observed outcome correlates with the results reported in previous studies, which have revealed a reduction in EE linked to lipid content.^61^

### 3.3. SEM analysis and particle size and zeta characterization

**Fig. 3:**
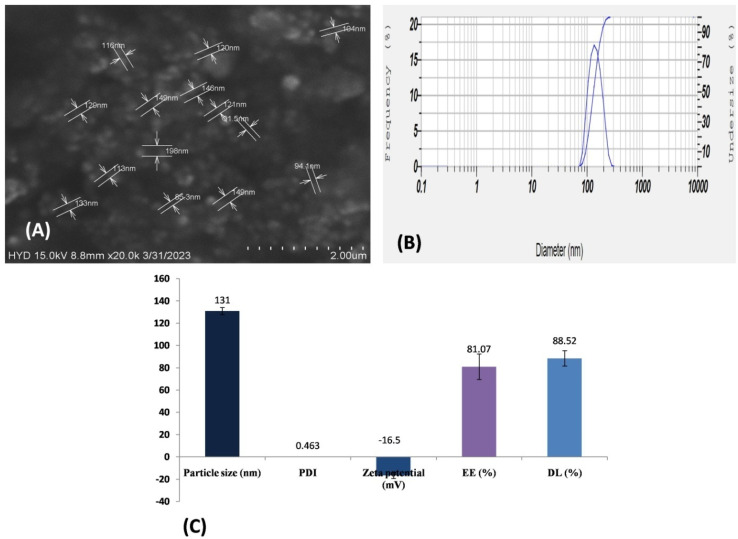
(A) Selected formulation (NP7) in SEM observation; (B) particle size determination of NP7 by laser light scattering technique denoted 131.0 nm diameter; (C) bar diagram presentation of promising NP7 SLN highlighting PDI, zeta potential, EE and DL.

The light scattering method was employed to determine the particle size, which ranged from 120.7 nm to 131.0 nm. NP1 displayed the smallest particle size (120.7 nm), while NP7 exhibited the largest particle size, measuring 131 nm as noted in Fig. 1B. There was a positive connection observed between particle size and phospholipid concentration. The study is supported by Salem and colleagues, who observed a gradual augmentation in the size of nanoparticles in correlation with the phospholipid concentration.^62^ In the same direction, Khan *et al.*,^63^ observed that the particle size of nanosized transferosomes increased proportionally with enhance of phospholipid content. The SEM images depicted in this research exhibit a dispersed arrangement of the SLN within the dispersion for the most optimal formulation (NP7). The particles exhibited a notable degree of symmetry, as depicted in Fig. 3A. The inquiry into particle size was conducted on the best formulation (NP7), resulting 131.0 nm diameter (Fig. 3B). The value of the polydispersity index (PDI) was determined to be ranged from 0.375 to 0.463. This implies that all developed SLNs have a uniform distribution.

The zeta value is commonly acknowledged as a dependable measure of the extent of dispersion and stability by the nanodosage form. A SLN that exhibits a greater zeta potential value, ideally surpassing ±10 mV, indicates a favorable and stable characteristic. On the other hand, a decreased zeta number indicates a tendency towards aggregation and flocculation. The findings indicated a notable alteration in the zeta value of SLNs. The positive zeta potentials of NP1 and NP2 were observed to be +6.3 mV and +4.2 mV, respectively. These values exhibited a progressive drop, followed by an increase in negative zeta potential. The NP3 demonstrated a zeta potential of −8.5 mV, while the NP7 displayed a zeta potential of −16.5 mV (Fig. 3C). The positive zeta value may be attributed to the presence of an amino group on NTC,^64^ which undergoes a slow transition to a negative charge upon binding with negatively charged LPC, namely through the interaction with the negatively charged phosphate group. In reference to the aforementioned statement, a study conducted by Iswanti *et al.*,^65^ in 2019 yielded comparable findings. The researchers observed that chitosan nanoparticles, which initially exhibited a positive surface charge, underwent a change to a negative charge upon interacting with cytosine–phosphate–guanine oligodeoxynucleotides.

### 3.4. ^1^H-NMR spectroscopy interpretation

**Fig. 4:**
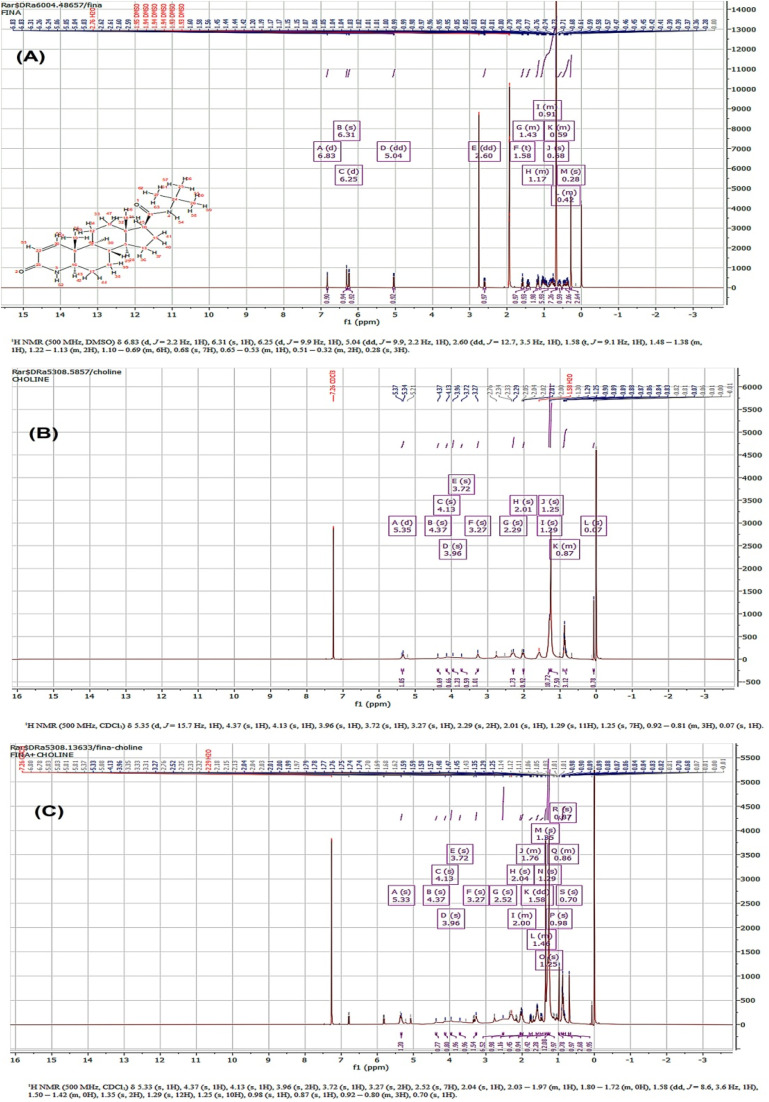
^1^H-NMR spectra observation of (A) FINA information represented as *δ* 6.83 (d, *J* = 2.2, 1H), 6.31 (s, 1H), 6.25 (d, *J* = 9.9 Hz, 1H), 5.04 (dd, *J* = 9.9, 2.2 Hz, 1H), 2.60 (dd, *J* = 12.7, 3.5 Hz, 1H), 1.58 (t, *J* = 9.l Hz, 1H), 1.48–1.38 (m, 1H), 1.22–1.13 (m, 2H), 1.10–0.69 (m, 6H), 0.68 (s, 7H), 0.65–0.53 (m, 1H), 0.5–0.32 (m, 2H), 0.28 (s, 3H); (B) LPC information represented as *δ* 5.35 (d, *J* = 15.7 Hz, 1H), 4.37 (s, 1H), 4.13 (s, 1H), 3.96 (s, 1H), 3.72 (s, 1H), 3.27 (s, 1H), 2.29 (s, 2H), 2.01 (s, 1H), 1.29 (s, 11H), 1.25 (s, 7H), 0.92–0.81 (m, 3H), 0.07 (s, 1H); (C) selected SLN (NP7) the data appears as *δ* 5.33 (s, 1H), 4.37 (s, 1H), 4.13 (s, 1H), 3.96 (s, 2H), 3.72 (s, 1H), 3.27 (s, 2H), 2.52 (s, 7H), 2.04 (s, 1H), 2.03–1.97 (m, 1H), 1.80–1.72 (m, 0H), 1.58 (dd, *J* = 8.6, 3.6 Hz, 1H), 1.50–1.42 (m, 0H), 1.35 (s, 2H), 1.29 (s, 12H), 1.25 (s, 10H), 0.98 (s, 1H), 0.87 (s, 1H), 0.92–0.80 (m, 3H), 0.70(s, 1H).

The structure of FINA, LPC and selected formulation (NP7) characterized by ^1^H-NMR spectroscopy. The Fig. 4A shows the spectrum of FINA. A high intensity singlet *δ* value of 6.83, corresponding to CO–CH, was discovered. Similarly, a singlet *δ* value of 6.31, relating to –NH, was also noted. It is widely recognized that electronegative atoms, such as oxygen and nitrogen induced deshielding of protons, resulting in downfield absorption. The degree of deshielding, as indicated by the *δ* value is directly correlated with the electronegativity of the atoms and their proximity to the proton.^66^ A prominent peak with high intensity was seen at a chemical shift of *δ* = 2.6, which can be attributed to the presence of =NH. Additionally, a triplet with a chemical shift of *δ* = 1.58, corresponding to 3H (CH_3_), was also identified.

The Fig. 4B shows the ^1^H-NMR spectrum of LPC. Structurally it is a class of phospholipids that incorporate choline as a headgroup. The ^1^H-NMR spectrums revealed a high intensity singlet *δ* value of 1.29 which is corresponding to 11H (CH_3_), similarly singlet *δ* value of 1.25 noted corresponding to 7H (CH_2_) observed. A small intensity but downfield peak appeared at *δ* = 4.37 as a result of O–H.

During the course of the investigation, an analysis was conducted to examine the molecular-level structure of FINA-LPC chosen NP. The objective was to investigate the potential effects of phosphatidylcholine in the formulation. According to the information presented in the Fig. 4C. It is observed that the NPs exhibit distinct, well-defined, and strong signals of the phosphatidylcholine protons in the range of 0.70 to 5.33 *δ*. The signals exhibited by the NPs containing phosphatidylcholine demonstrate a moderate intensity and breadth. Nevertheless, a limited number of significant peaks were observed in the upfield NMR spectrum within the range of 0.70 to 1.46 *δ*. The noticeable peak of FINA is identified and assessed in a systematic manner. A *δ* value of 1.58, which is mostly attributed to the presence of the –CH_3_ group in the FINA molecule, was detected. Furthermore, the value of *δ* = 1.29, which corresponds to 12H (CH_3_), indicates the presence of l-α-phosphatidylcholine. Interestingly, the peak corresponding to the presence of =NH, which was highly prominent (*δ* = 2.6) in the first pure FINA ^1^H-NMR spectra, was unexpectedly absent in the current analysis. Similarly, the strong peaks for CO–CH and –NH were not found in the selected sample. It is widely recognized that protons attached to the –NH group exhibit significant exchangeability, resulting in broad signals or potential disappearance in NMR spectra,^67,68^ particularly in solvents that promote this equilibrium. It can be inferred that the proton may have been involved in bond formation in the NP formulation, and the larger concentration could facilitate the observation of broader signals.

### 3.5. ATR interpretation

**Fig. 5:**
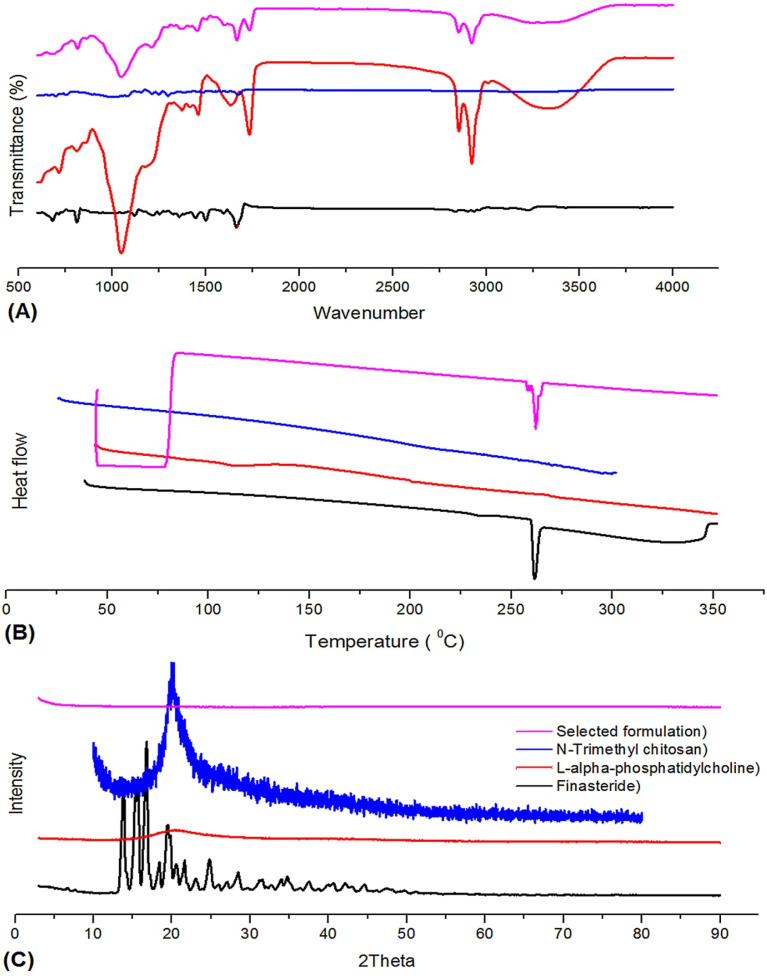
(A) ATR spectra construction of FINA, LPC, NTC and selected formulation (NP7). A significant and pronounced band was observed at a wavenumber of 1666.62 cm^−1^, which represent the vibration of the C=O bond by stretching. The distinctive peak associated with –NH stretching was not seen; (B) DSC thermogram revealed that the NP7 exhibited an endothermal peak at 259.84 °C. The presence of a sharp peak suggests the existence of stable FINA; (C) XRD spectra displayed a decline in peak intensity, indicating a decrease in the crystallinity of FINA within the SLN (NP7).

FINA is a chemically synthesized polycyclic steroid compound that serves as an analog of androgenic steroid hormones, such as testosterone. The molecule consists of two N–H groups situated on the cyclic ring. The spectral region where the stretching vibrations of the N–H bonds are observed is typically within the range of 3600–3300 cm^−1^. The N–H stretching was seen at 3425.11 cm^−1^. Likewise, N–C stretching vibrations are designated at a wavenumber of 811.51 cm^−1^. One of the most distinctive attributes of the carbonyl group is the presence of a distinct peak, typically detected within the wavelength range of 1850–1550 cm^−1^. The ATR spectrum exhibits a prominent and intense band at 1664.60, corresponding to the C=O stretching vibration as seen in Fig. 5A. This particular vibration is attributed to the presence of a carbonyl group.^69^ The experimental spectrum reveals the presence of the C=C stretching vibration of FINA at a wavenumber of 1500.45 cm^−1^, exhibiting a band of significant intensity. Characteristic C–C stretching modes were discovered at 1219.59 cm^−1^. It is known for C–C bending vibration in FINA induces skeletal elongation of the ring structure. The frequency observed at 681.50 cm^−1^ is commonly associated with the bending vibrations of carbon–carbon (C–C) bonds inside the ring structure. The unsaturated C–H stretching vibration is designated at a wavenumber of 3224.11 cm^−1^ at higher frequencies.

Figure displays the measured distinctive frequency peaks of LPC. The observed spectral band is located at approximately 3346.57 cm^−1^, and it can be ascribed to the CH mode of the methyl groups coupled to nitrogen (choline). Additionally, notable CH bands are observed at around 2922.95 cm^−1^. A prominent peak was observed at a wavenumber of 1047.17 cm^−1^, which can be attributed to the stretching vibration of the P=O bond in the phosphate group.^70^ Likewise, it was observed that there was a clear peak at 1734.13 cm^−1^, which is responsiblefor a carbonyl (C=O) group.

The NTC ATR spectra exhibited prominent peaks at a wavenumber of 677.11 cm^−1^, corresponding to the out-of-plane bending motion of the C–O bond. The out-of-plane bending of the NH moiety exhibited a maximum intensity at a wavenumber of 749.87 cm^−1^. In the same way, significant peaks were seen at wavenumbers of 1232.16 cm^−1^ and 2962.51 cm^−1^, corresponding to the stretching vibrations of C–O–C and CH_2_ groups, respectively. Furthermore, a distinct peak was seen at 3542.09 cm^−1^, indicates –OH stretching. A prominent peak was seen at 1722.47 cm^−1^, which corresponds to the stretching of amide group. It noted NTC compound exhibited absorption bands at wavenumbers 2918.41 cm^−1^ and 2851.06 cm^−1^, corresponding to the symmetric and asymmetric stretching of C–H bonds, respectively. Furthermore, it is worth noting that a narrow and highly concentrated spectral peak at a wavenumber of 1547.19 cm^−1^ has been observed, which is attributed to the amine group (N–H bending) that is found in NTC.

The selected formulation was subjected to ATR and meticulously assessed to detect any possible interactions. These interactions were then highlighted. The N–C stretching vibrations are observed at a specific wavenumber of 815.21 cm^−1^. The experimental spectrum demonstrates the existence of the C=C stretching vibration in the optimal formulation, observed at 1501.07 cm^−1^. The designation of a broader peak at a wavenumber of 3301.32 cm^−1^ can be designated to the unsaturated C–H vibration (stretching). Additionally, we observed a presence of C–C stretching band associated with the ring structure in the most optimal solid lipid nanoparticle (SLN). The wavenumber of 1048.84 cm^−1^ was observed as a peak, and it was determined that this peak attributedby the vibration (stretching) of the P=O bond in LPC. Nevertheless, it is important to acknowledge that the distinctive peak associated with –NH stretching was not seen. The observed contact can be ascribed to the reaction between protons connected to the –NH group and the positively charged choline group of LPC.^71,72^ The information presented is substantiated by the results obtained from the previously mentioned examination of ^1^H-NMR spectra.

### 3.6. DSC interpretation

The DSC research revealed the existence of an endothermic peak occurring at a temperature of 261.43 °C while examining pure FINA. The presence of a distinct peak in the data suggests, based on the relevant literature of high purity (Fig. 5B). It noted a distinct peak with a wide base is visible at a temperature of 83.36 °C, suggesting the high degree of purity and the existence of LPC. However, it is worth noting that there was an extra-wide endothermal peak observed at a temperature of 196.87 °C. This peak could perhaps be attributed to the thermal digestion process, namely the denaturation of lipids, which occur at high temperatures.^73,74^ NTC is an excipient derived from chitosan through a chemical procedure. Chitosan is derived from a diverse range of marine exoskeletal organisms. In our investigation three distinct endothermal peaks were seen at temperatures of 214.06 °C, 271.24 °C, and 278.39 °C, indicating the NTC originating from natural sources. The findings derived from the DSC investigation revealed that the best formulation exhibited an endothermal peak at 259.84 °C, depicted in Fig. 5B. The presence of a sharp peak suggests the existence of stable FINA. However, the smaller intensity of the peak indicates a potentially lower rate of transformation of FINA with low enthalpy.^75^ Additionally, it was observed that there is a wider peak at 77.53 °C, which suggests the occurrence of a semi-crystalline polymeric transition in FINA-LPC SLNs.

### 3.7. XRD interpretation

The primary objective of XRD study is to acquire a distinguishable and well-defined peak that arises from an object exhibiting a high level of crystallization, while simultaneously reducing any potential errors caused by background noise. In the current study, the crystallographic pattern and changes in the drug following its conjugation with different copolymers and chemicals were assessed using XRD analysis. The sample of FINA exhibited distinct peaks at 13.77 (2*θ* degree), 15.33 (2*θ* degree), 16.71 (2*θ* degree), 19.52 (2*θ* degree), and 24.80 (2*θ* degree), with an intensity below 4000. Fig. 5C illustrates the presence of supplementary peaks with intensities below 1100 at 20.55, 21.56, 28.44, and 33.94 (2*θ* degree), among others. This confirms higher crystalline nature of FINA. The XRD image displayed a wider and more intense peak observed at 20.41 (2*θ* degree), which is attributed to LPC. The broadening of the peak can be ascribed to a decrease in crystallinity, potentially resulting from a greater amount of long-chain saturated fatty acids.^76^ It ascertained a notable peak at 22.31 (2*θ* degrees) with an intensity below 350 for NTC. Additionally, numerous smaller peaks were observed, suggesting the potential existence of moisture in the NTC and a significant level of background noise.^77^ Based on the data depicted, the best formulation exhibited a limited number of noticeable peaks at 61.94 and 81.63° (2*θ*), along with numerous peaks of lower intensity. Additionally, it was observed that the diffractogram displayed a decline in peak intensity, indicating a decrease in the crystallinity of FINA within the SLN.

### 3.8. Stability study of selected formulation

Stability experiments for the best formulation were performed under accelerated conditions in accordance with International Conference on Harmonization requirements (ICH) guideline. For the storage of the optimal formulation, a well-closed container or a sealed vial was utilized. The vial contains “NP7”, which was stored in a stability chamber (Stability models 3907, Thermofisher, Mumbai). Gel formulations were stored under the conditions stated in Table 1 for the duration given. The formulation's particle size, PDI and zeta potential were assessed. The information narrated.

Stability testing information of NP7 as per ICH guideline

**Table:** 

Long term testing	Conditions: 25 °C ± 2 °C/60% RH ± 5%				
Evaluation parameter	Duration of study (month)				
	0	3	6	9	12
Particle size (nm)	131.0 ± 0.05	131.4 ± 0.01	132.6 ± 0.03	133.1 ± 0.02	133.9 ± 0.01
PDI	0.463 ± 0.01	0.468 ± 0.002	0.468 ± 0.002	0.473 ± 0.02	0.488 ± 0.001
Zeta potential (mV)	−16.5 ± 0.2	−16.9 ± 0.1	−16.2 ± 0.3	−15.8 ± 0.08	−15.8 ± 0.1

**Table:** 

Accelerated testing	Conditions 40 °C ± 2 °C/75% RH ± 5%		
Evaluation parameter	Duration of study (month)		
	0	3	6
Particle size (nm)	131.0 ± 0.05	131.3 ± 0.02	132.8 ± 0.01
PDI	0.463 ± 0.01	0.467 ± 0.002	0.469 ± 0.001
Zeta potential (mV)	−16.5 ± 0.2	−16.1 ± 0.1	−16.2 ± 0.07

The above table highlighted the impact of temperature and humidity on selected NP7 formulation. It observed no such significant changes for particle size, PDI and zeta potential noted in long term and accelerated stability study. The study confirmed the stability of our developed formulation.

### 3.9. Permeability and skin retention observation

**Fig. 6:**
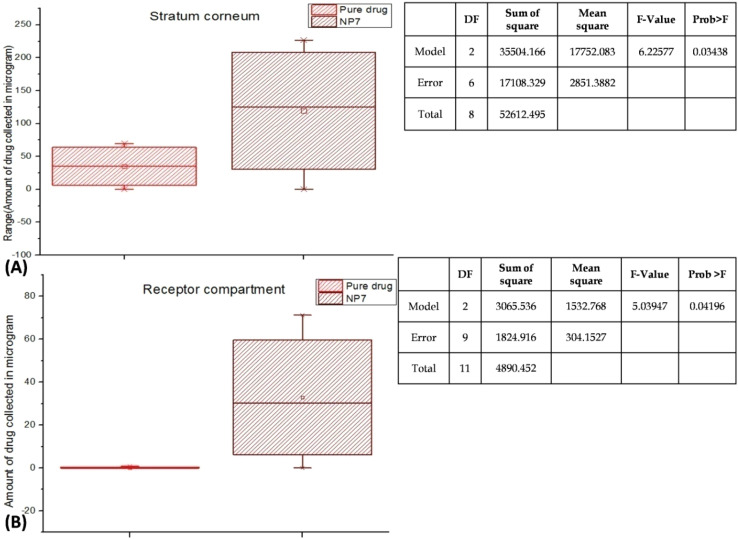
(A) Drug retention on SC layer by NP7. The box-plot chart and study illustrates the retention of FINA, including both NP7 and the pure drug, within the SC layer. NP7 exhibited a notable retention of drug 226.76 μg at 18 h. The study supported by one way ANOVA (*p* < 0.05), which shows significant result; (B) FINA permeated in receptor chamber by Franz diffusion study. The receptor compartment exhibited a modest quantity of FINA from NP7 (71.23 μg) over 18 h which is supported by one way ANOVA (*p* < 0.05).

The study illustrates the retention of FINA, including both NP7 and the pure drug, within the SC layer during a designated duration of time. According to the statistics, the initial concentration of FINA in the first 6 h of the study was 12.92 μg, which climbed to 69.73 μg after 18 h. In contrast, it was observed that NP7 exhibited a notable retention of drug 226.76 μg for duration of 18 h. The findings indicated that the levels of FINA derived from the NP7 were significantly elevated compared to those obtained from the pure drug. The observed substaposition of FINA on the SC may be attributed to the presence of negatively charged lipids,^78^ which facilitate the deposition of a significant quantity of charged drug from NP7-SLN (Fig. 6A). Similarly, adhesive nature of NTC executed better bioadhesion which facilitated for extended drug release. The assertion made is corroborated by the findings of Lin H. *et al.*,^79^ who observed the presence of charges on lipid vesicles contributes to the enhancement of skin permeability. Maione-Silva L. *et al.*,^80^ conducted a study in which they observed that ascorbic acid-loaded liposomes with negative charges exhibited increased permeation and retention than those with positive charges. The researchers documented a 62% increase in flux due to surface negativity and a 600% rise in drug retention compared to unmodified ascorbic acid. The observed enhancement and increased capability of FINA in retaining SC is likely attributable to the capacity of LPC to induce temporal changes in the epidermis layer, as demonstrated in our study.^81^ Moreover, LPC have the potency to stop the *trans*-epidermal water loss which hydrate SC layer and ultimately favor for greater retention of FINA. The results of this study demonstrated that LPC when used as a carrier for FINA, exhibited effective dispersion throughout the dermis. This finding highlights the potential benefits of utilizing LPC as a vehicle for drug delivery, as it allows for optimal drug accumulation as well as controlled release.

Remarkably, the presence of FINA in the receptor compartment was unnoticed from pure drug (Fig. 6B). Nevertheless, the receptor compartment exhibited a modest quantity of FINA from NP7 (71.23 μg) over 18 h. The aforementioned study was supported by Kim C. *et al.*,^82^ as they demonstrate a notable increase in the epidermal retention and permeability of caffeine when Phosphatidylcholine is present. Similarly, in a study conducted by Lv x. *et al.*,^83^ it was observed that vitamin C exhibited significant permeability and retention efficiency on the skin when formulated with Phosphatidylcholine.

### 3.10. Hair growth by SLN in C57BL/6 mice

**Fig. 7:**
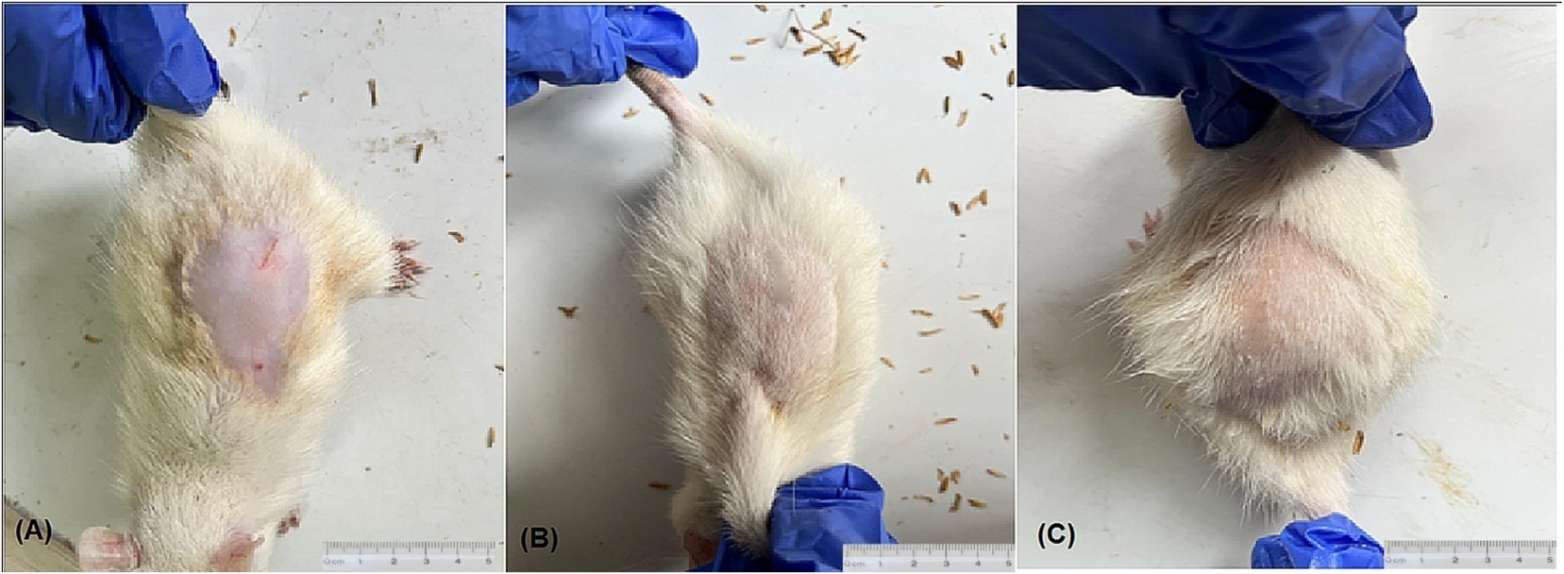
(A) Hair removal on the dorsal skin of C57BL/6 mice; (B) Group II treated with FIN XL gel; (C) Group IV treated with SLN-NP7. Both Group II and IV formulation had the favorable outcomes in terms of hair development, as well as considerably improved hair coverage and density.

To assess the efficacy of the formulated SLN in promoting hair regeneration, the hair growth of the AGA mice model was assessed through hair removal (Fig. 7A). Following this, a range of treatments were conducted. Periodic progressive hair development is observed on dorsal skin of C57BL/6 mice. In the present study, it was shown that both the positive control group (Group II FIN-XL gel, Fig. 7B) and Group IV (SLN-NP7, Fig. 7C) exhibited the most favorable outcomes in terms of hair development as well as considerably improved hair coverage and density. Nevertheless, the rate of growth observed in the Group IV population was comparatively lower than that shown in the Group II cohort treated with FIN-XL gel, yet it was higher than the growth rate observed in the Group III population alone administered with alone FINA (images in ESI file†). The data presented indicates stronger evidence on efficiency of hair growth by FINA in presence of LPC during the synthesis of SLNs without any visible cutaneous reaction on skin, as compared to the use of pure drug alone.

## 4 Conclusion

The ultrasonication technique was employed to include FINA in SLN along with LPC, and NTC. The observation revealed an uniform distribution of particles and symmetrical shape. The maximum size of 131 nm SLN was obtained by NP7, which exhibited the highest zeta potential value of −16.5 mV, indicating the successful stability of the selected SLN. The current research supports the theory that an increase in EE can be observed as the quantity of lipid (LPC) gradually increases from 50 to 150 mg. The results of the *in vitro* release analysis indicate that the drug release of “NP7” was the lowest, measuring 54.25% at 18 h, while maintaining the minimal effective concentration. The formulation designated as “NP7” was chosen as the optimal selection. The ^1^H-NMR spectra of the selected formulation displayed the absence of a prominent peak belonging to the =NH group, which typically occurs at a chemical shift (*δ*) value of 2.6. The ATR investigation demonstrated the occurrence of a reaction between proton that is bonded to the –NH group and the choline group of LPC, which has a positive charge. The DSC study observed a smaller peak in NP7, which was attributed to a decreased rate of transition of FINA with low enthalpy. One of the potential findings noted the investigation of SC retention and permeability by employing porcine ear skin. A noteworthy observation was made regarding the retention of FINA, with a significant amount of 226.76 μg being detected on the SC layer. In contrast, the permeability finding revealed a minimal quantity of FINA, measuring 71.23 μg, in the receptor chamber. Significant enhancements in hair coverage and density were found in Group IV, which was subjected to NP7 treatment, as depicted in Fig. 7 of the C57BL/6 mice model. The aforementioned discovery underscores the potential advantages of employing LPC as a means of administering drugs since it enables optimal drug accumulation and controlled release. The results also revealed a positive outcome in terms of hair growth, comparable to the group treated with FIN-XL gel. The study was performed in selected mouse model. However, the anatomical feature and function of mouse and human differences; this makes a challenging task to predict the human skin retention and hair growth. Furthermore, we are expecting few more studies for the long-term efficacy and safety of developed SLNs; such as carcinogenicity study, chronic toxicity study, pharmacokinetic study, organ specific toxicity, and genotoxicity in suitable animal model.

## Ethical statement

The study received ethical clearance; NCP/PhD/23-24/036.

## Data availability

The datasets generated and/or analyzed during the current study are available from the corresponding author upon reasonable request.

## Author contributions

All authors certify that they have participated sufficiently in the work to take public responsibility for the content, including participation in the concept, design, analysis, writing, or revision of the manuscript. HR: supervision, writing – original draft preparation, conceptualization, methodology, and investigation. BM: visualization, investigation and data interpretation. BSN: investigation, data curation, grammatical correction, figure construction. RAB: supervision, conceptualization and investigation.

## Conflicts of interest

The authors declare that they have no known competing financial interests or personal relationships that could have appeared to influence the work reported in this paper.

## Supplementary Material

RA-015-D5RA00399G-s001

RA-015-D5RA00399G-s002
