# Supplementary material for: Novel delivery strategy: finasteride-loaded solid lipid nanoparticles for improved androgenetic alopecia therapy
Source: RSC Adv. 2025 Jun 4;15(23):18715–31. doi: 10.1039/d5ra00399g (PMC12134744; doi:10.1039/d5ra00399g)

Supplementary Information (SI) for RSC Advances.
This journal is © The Royal Society of Chemistry 2025

Supporting supplementary images

Group III population alone administered with alone FINA

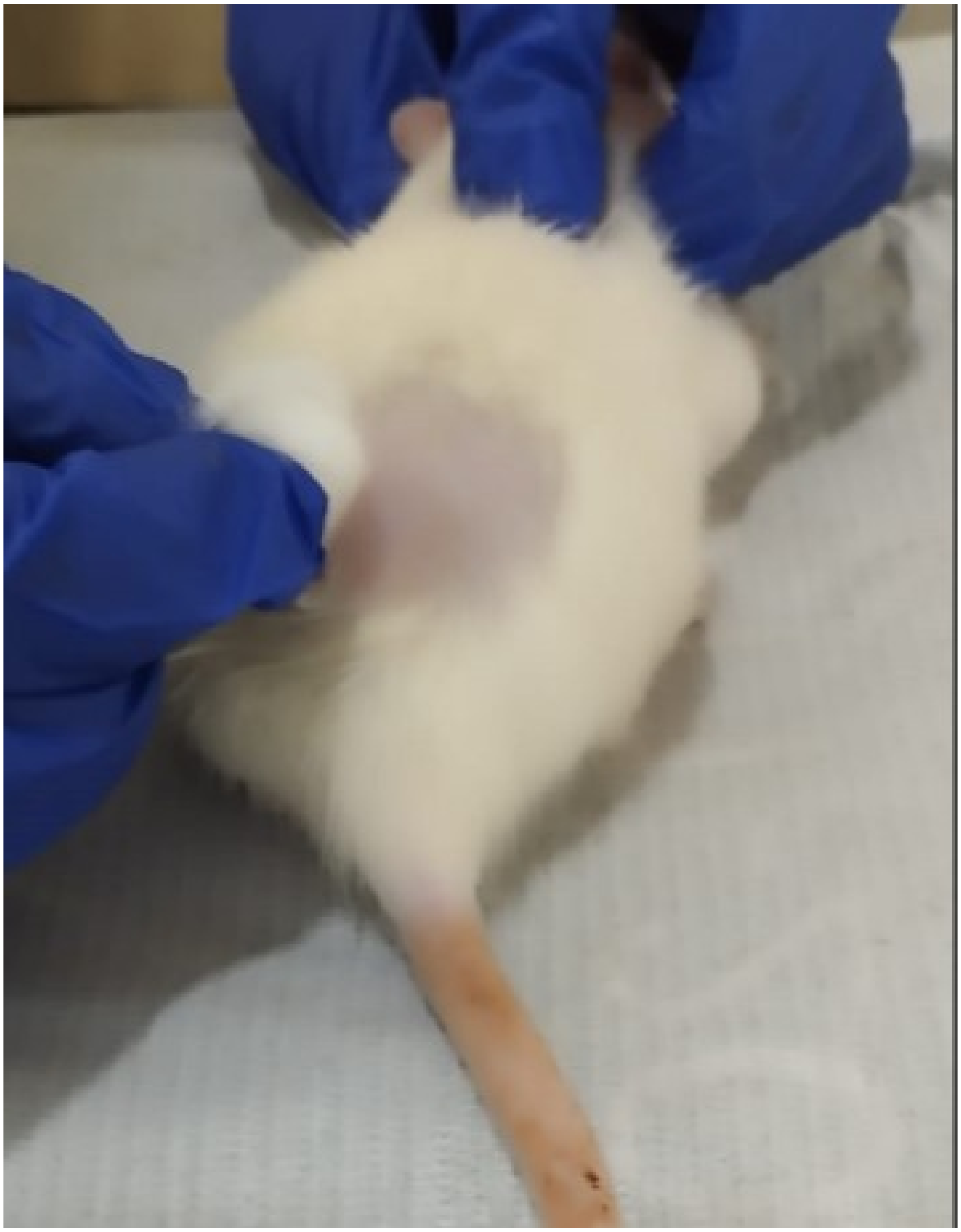

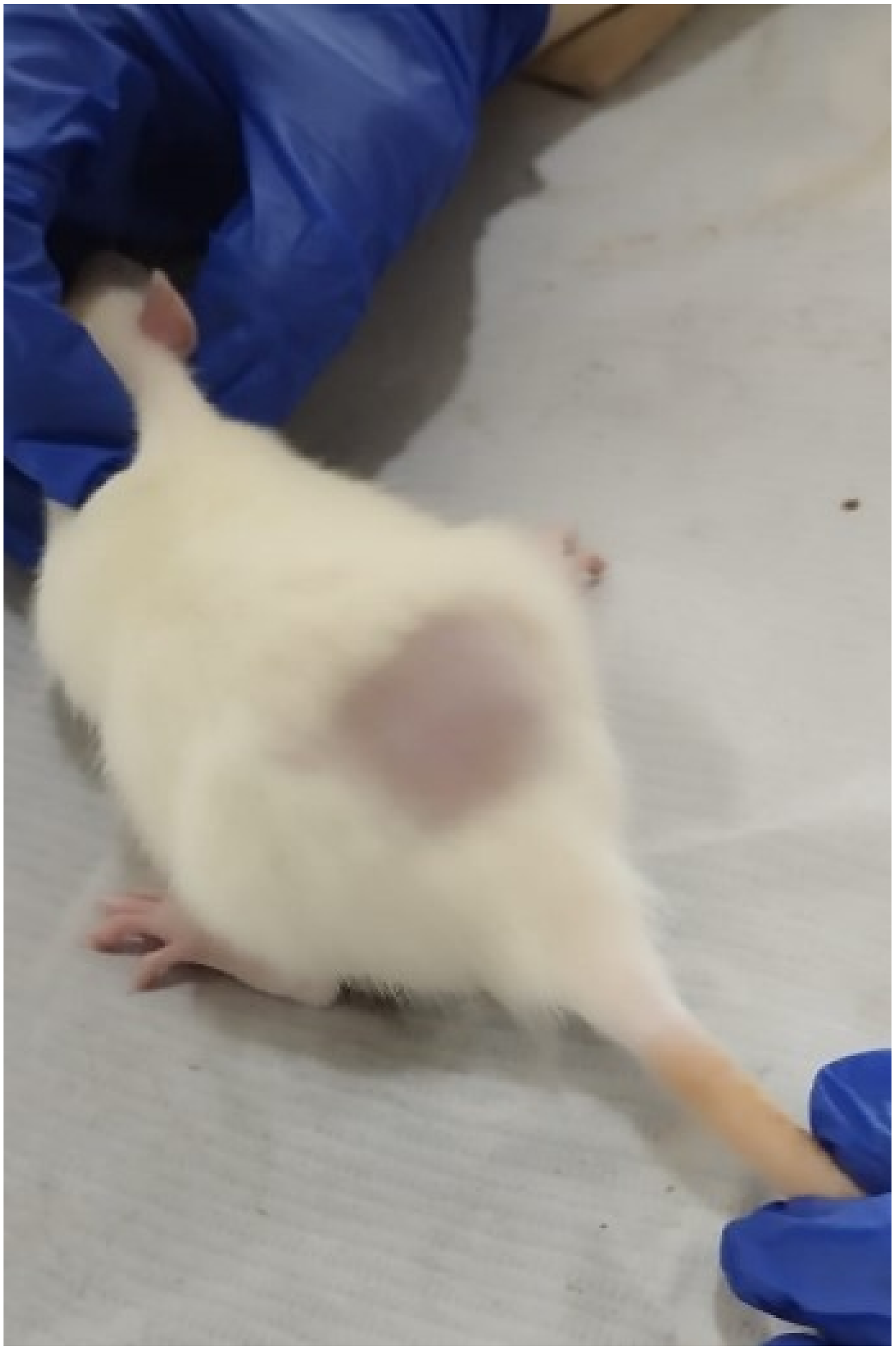

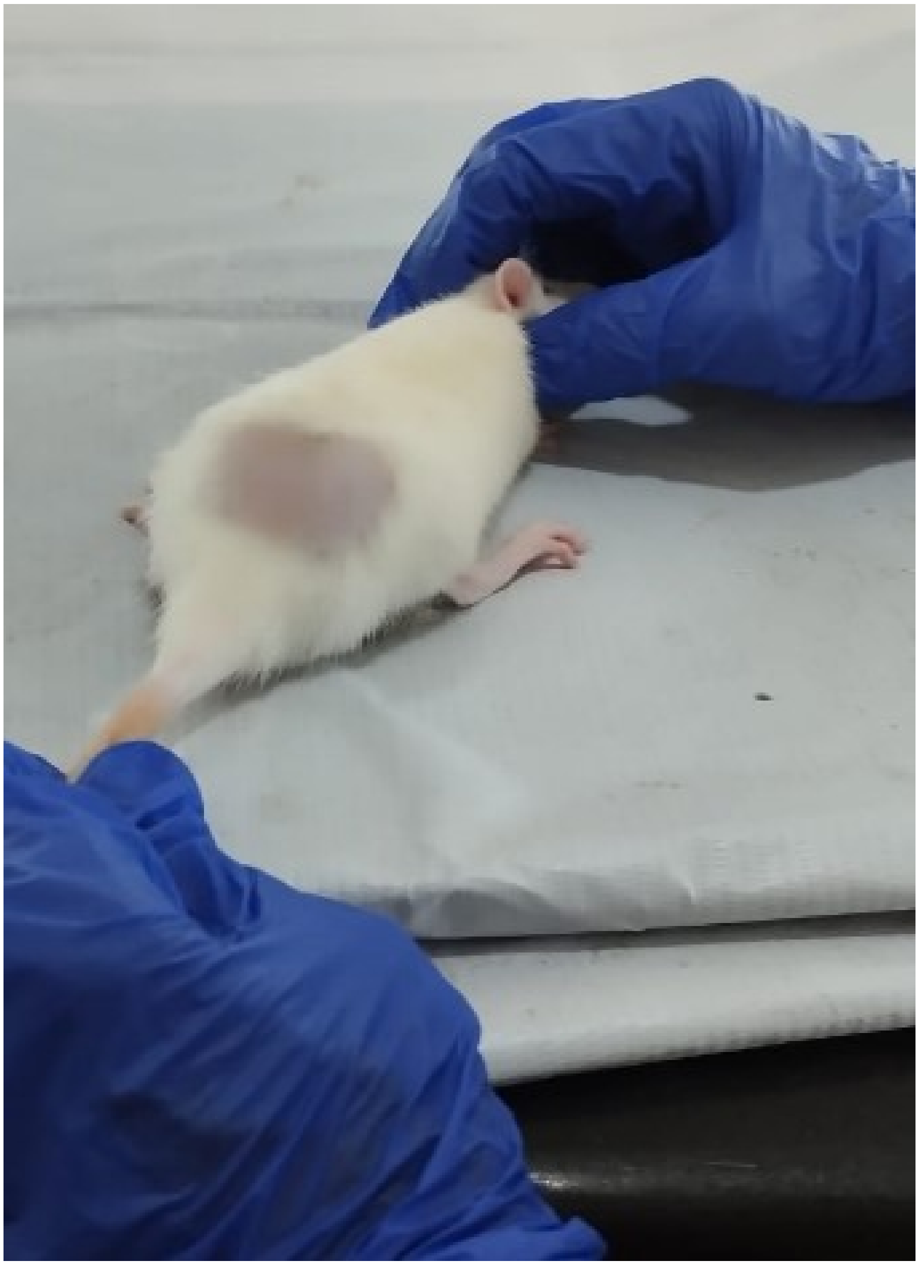

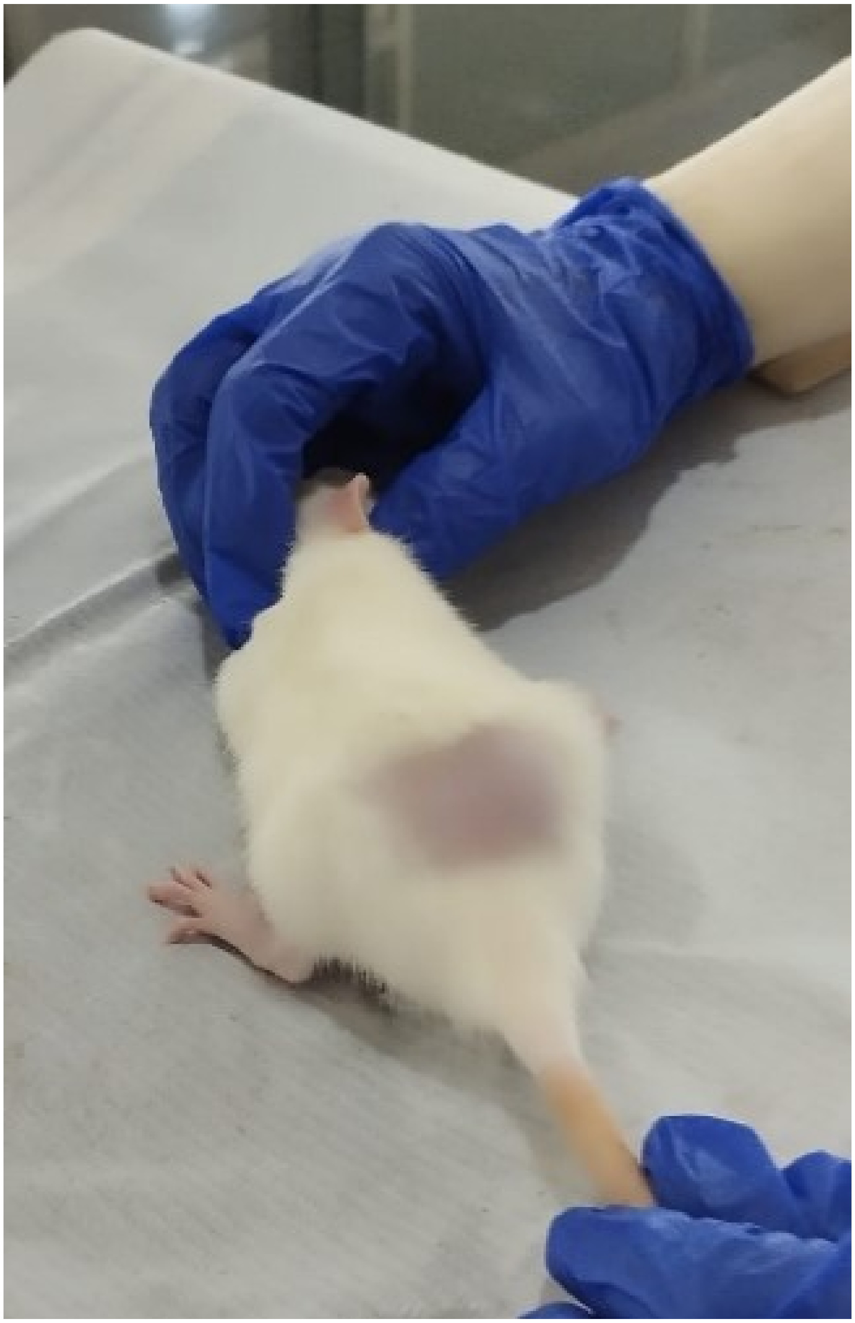

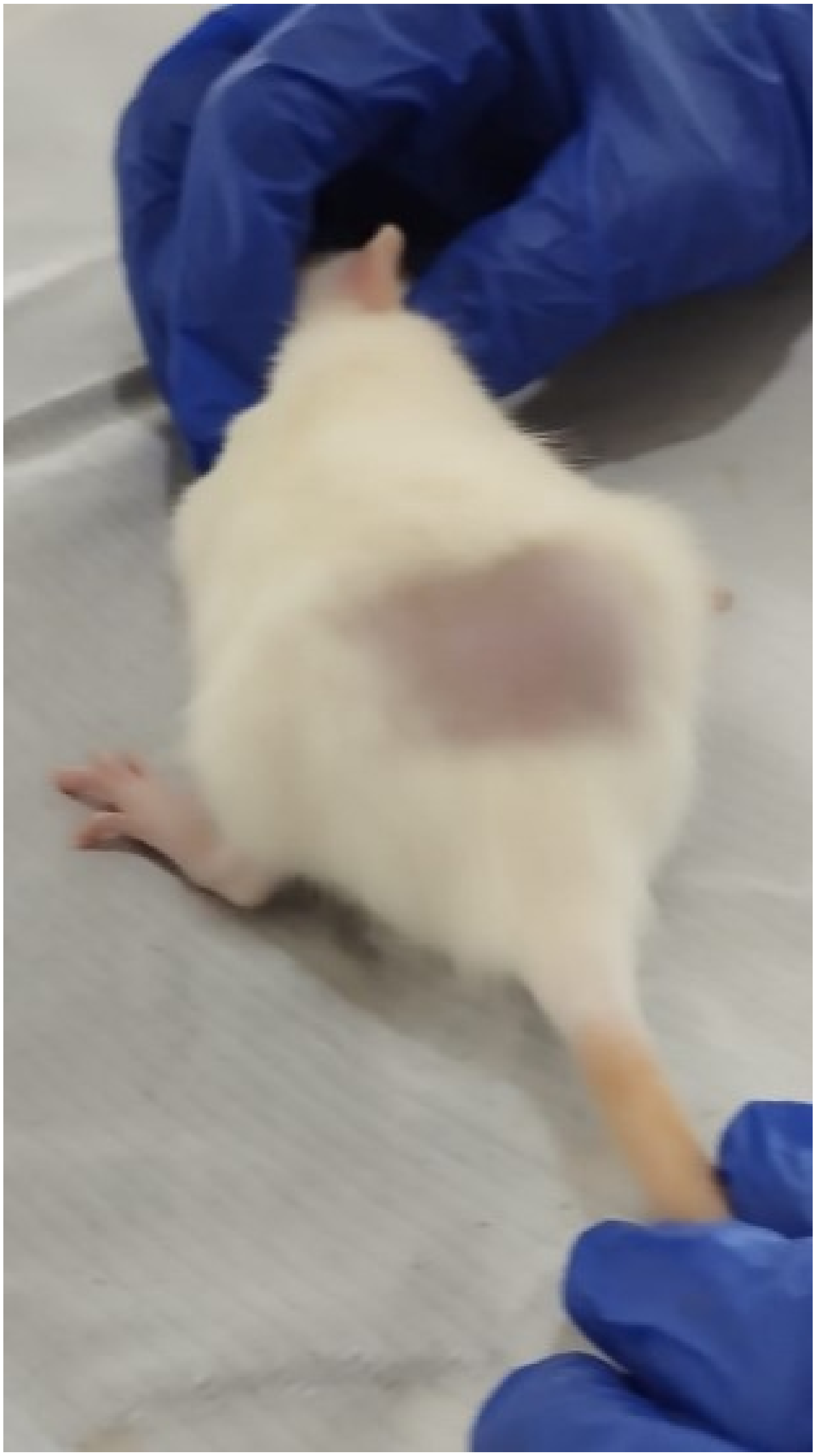

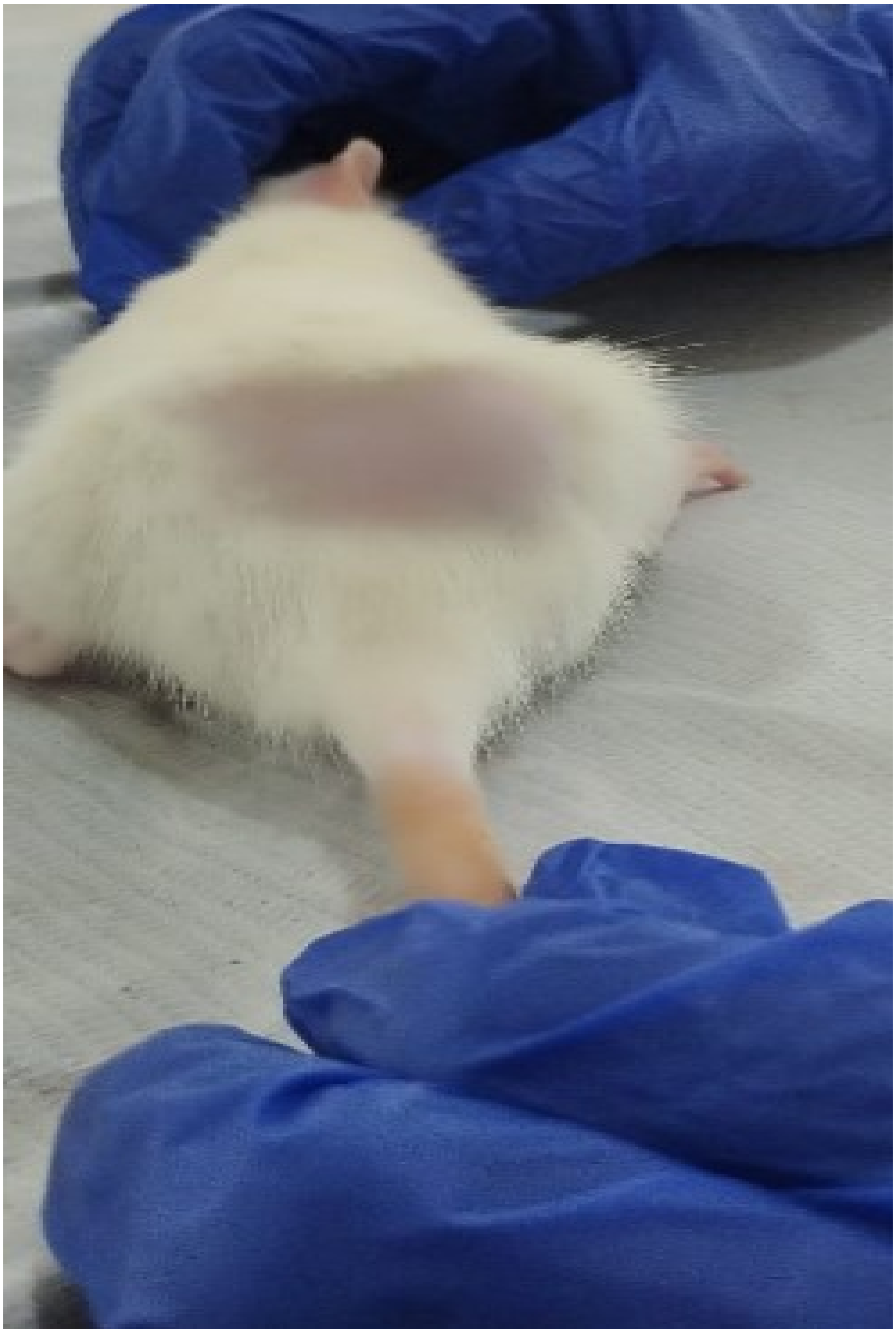

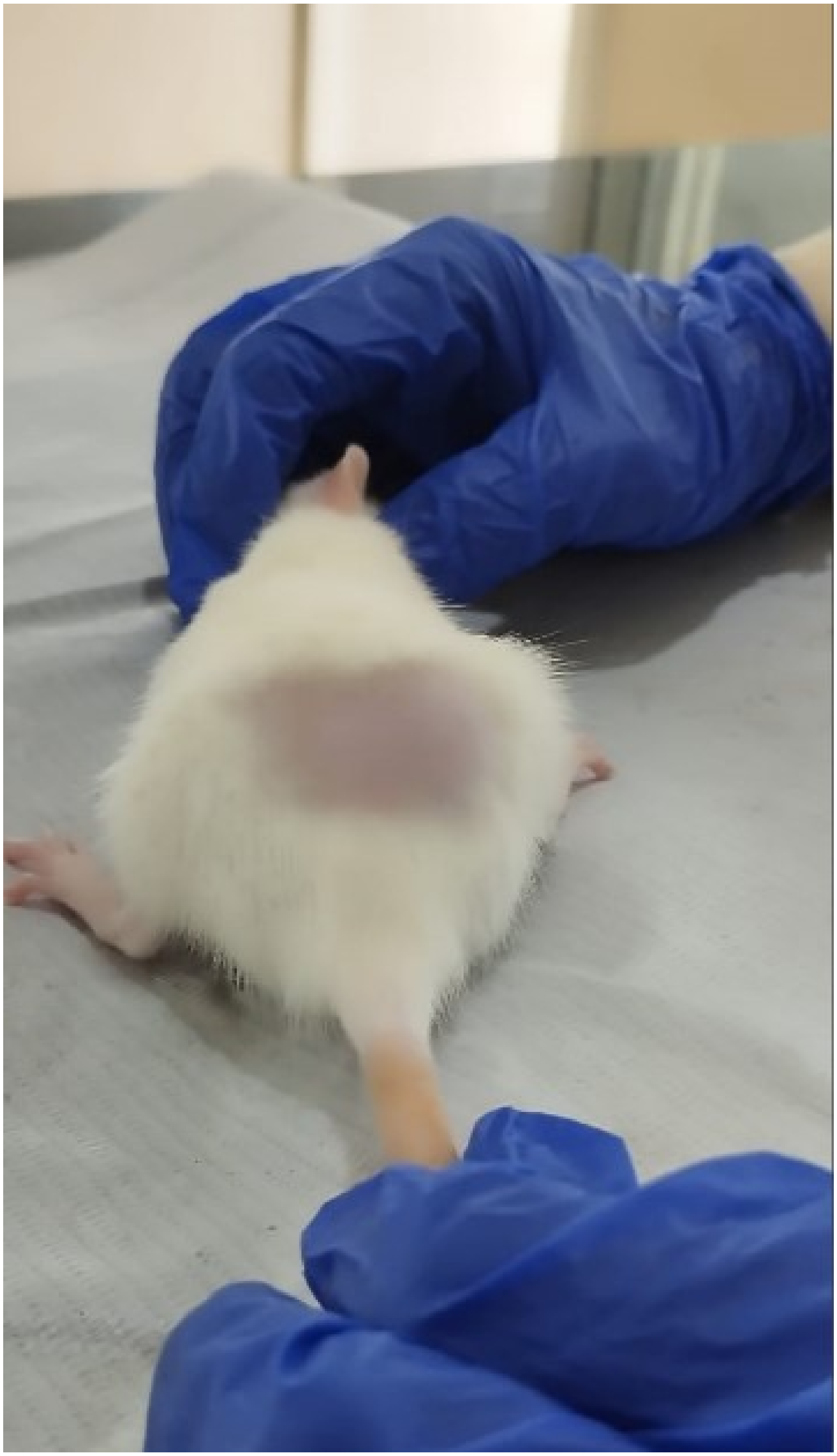

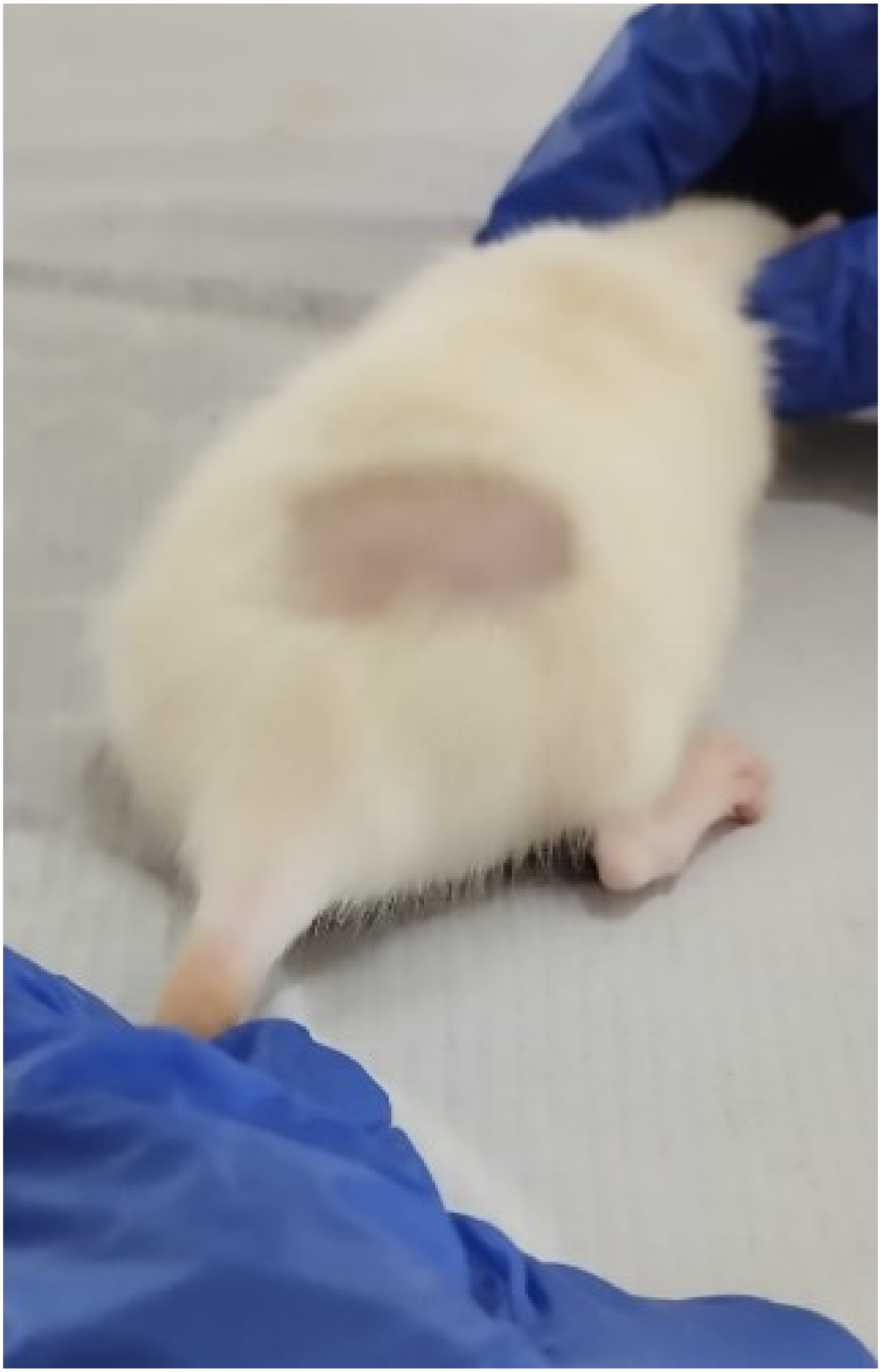

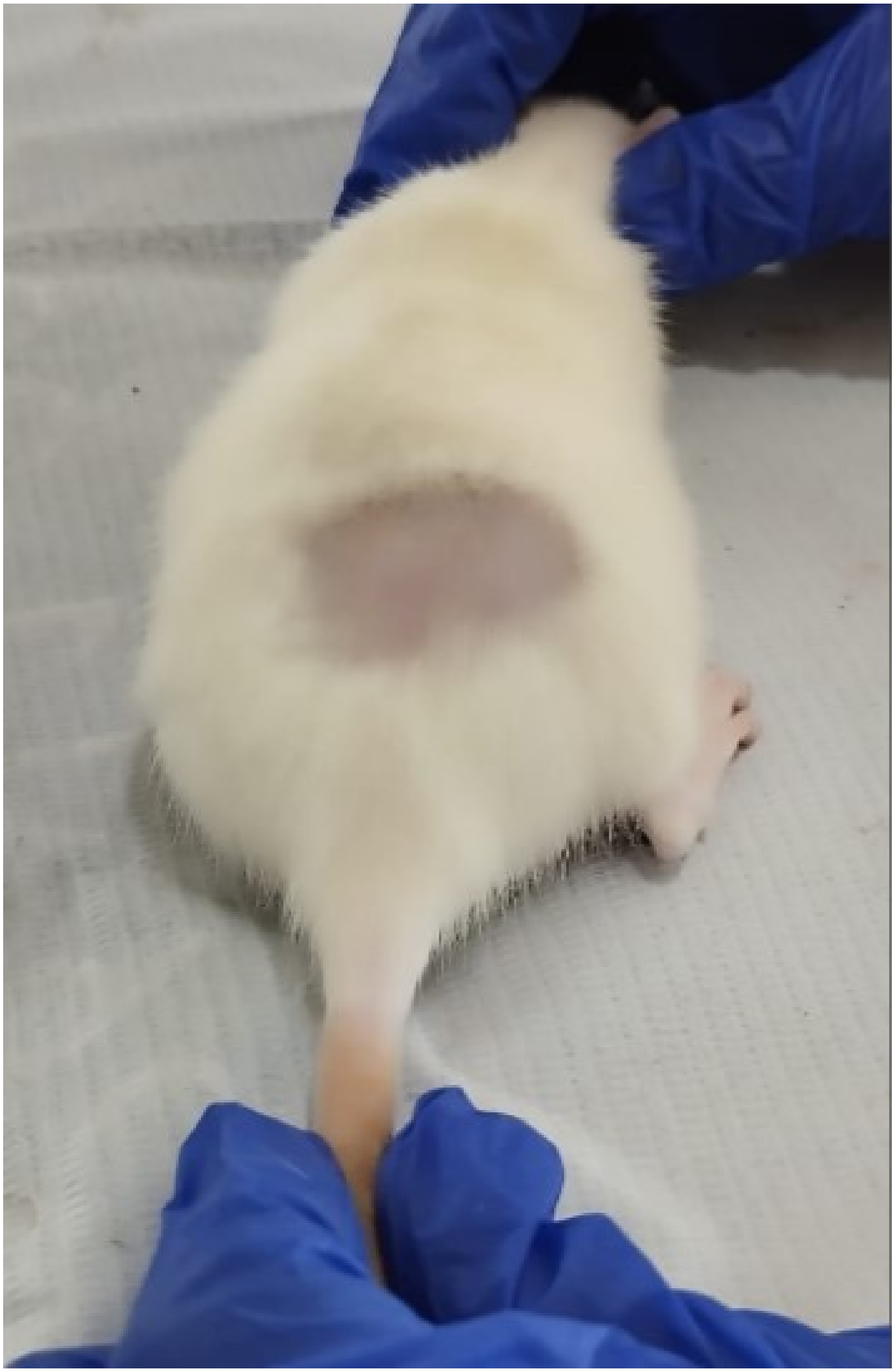

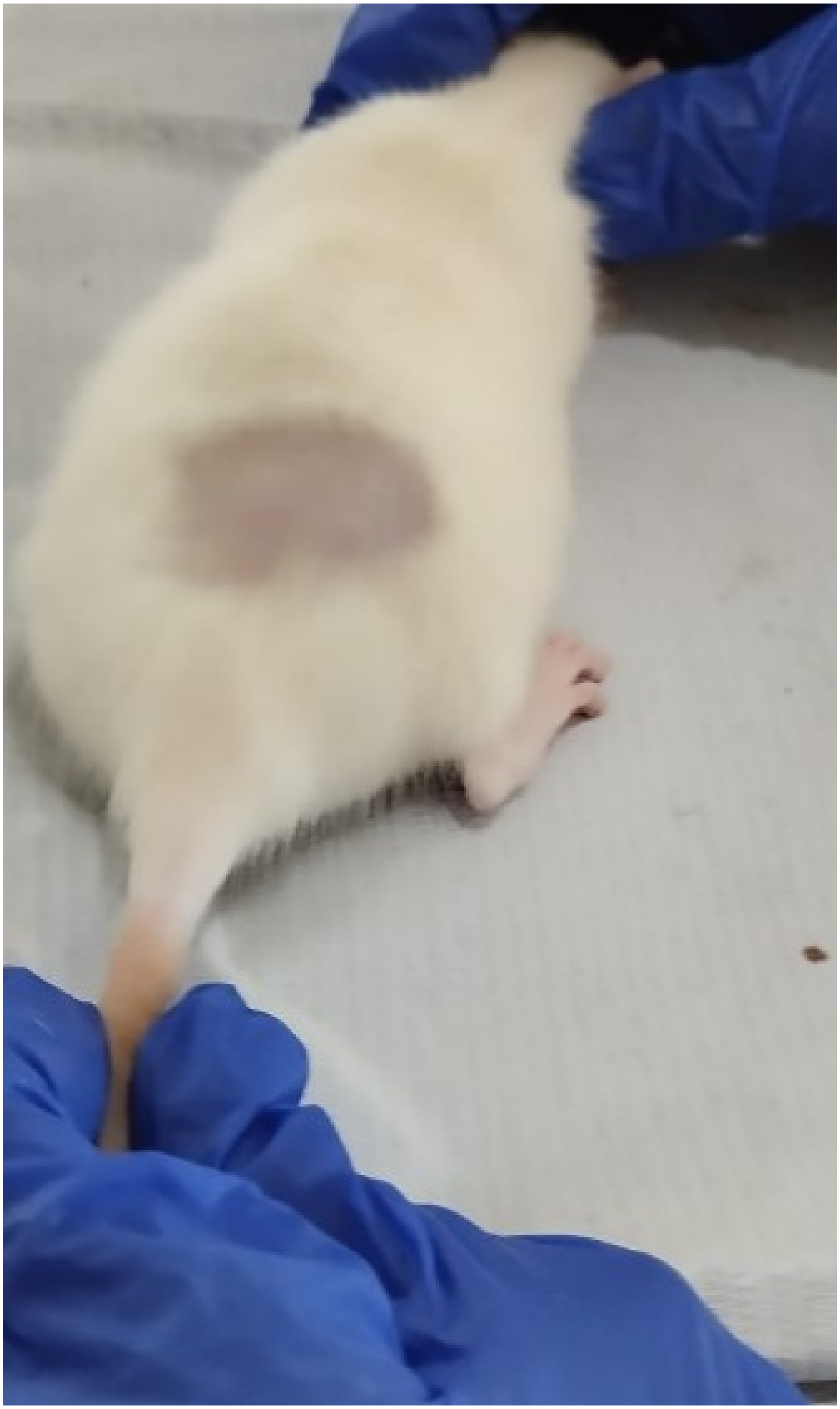

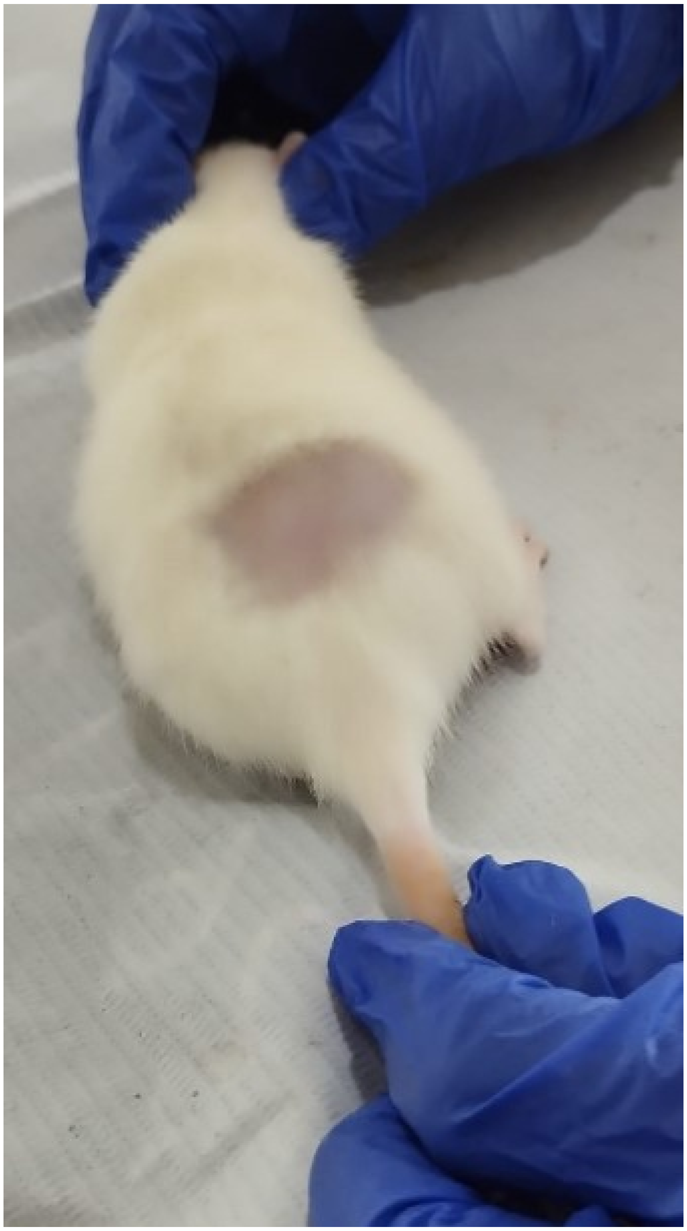

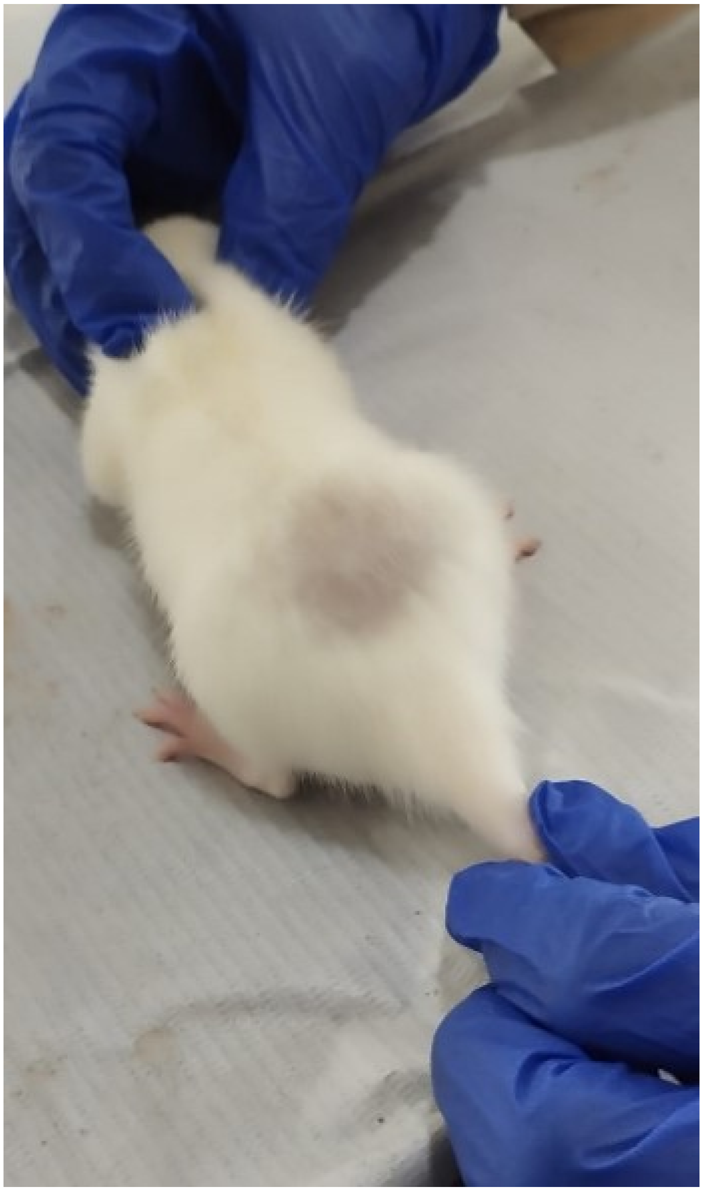

Group IV population treated with SLN-NP7

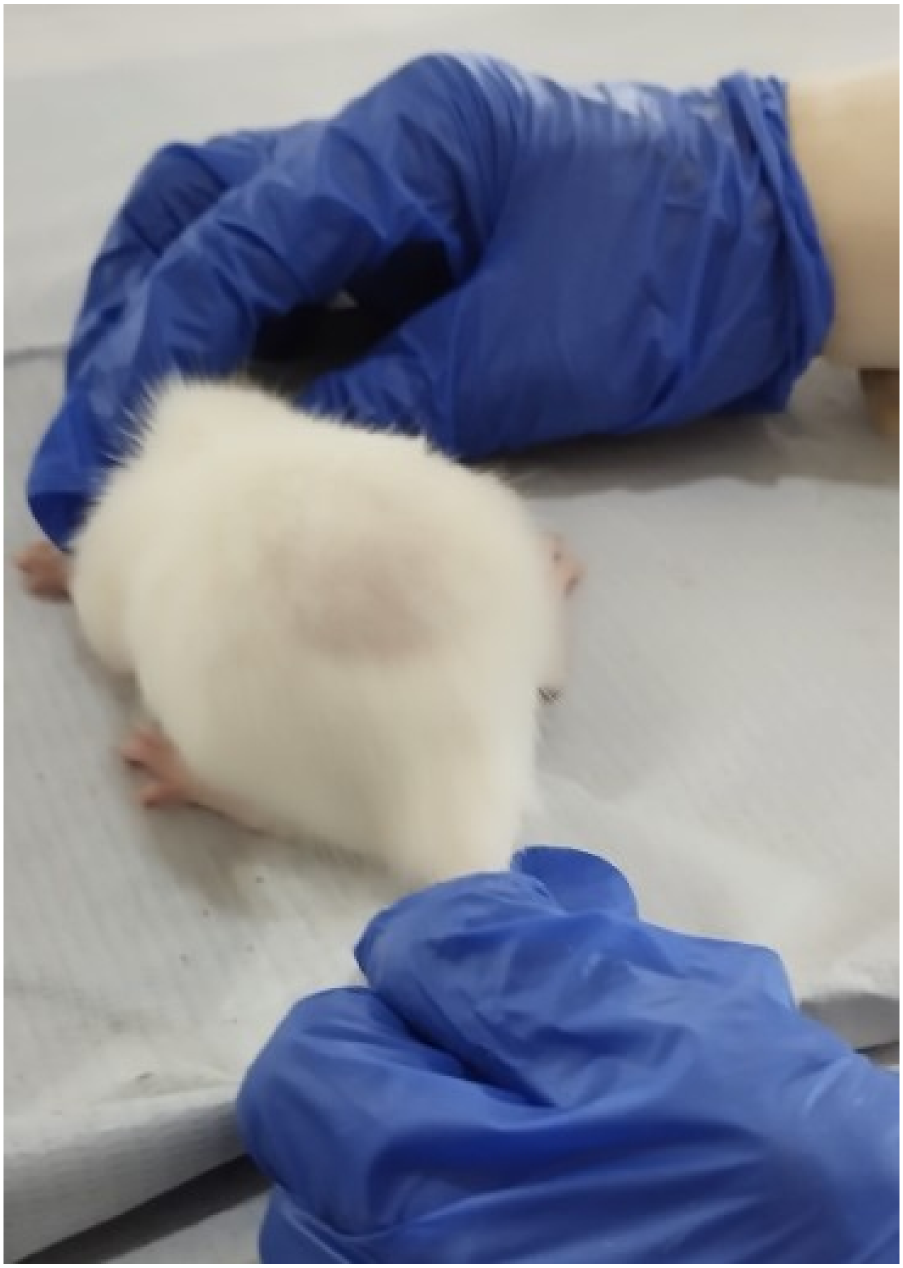

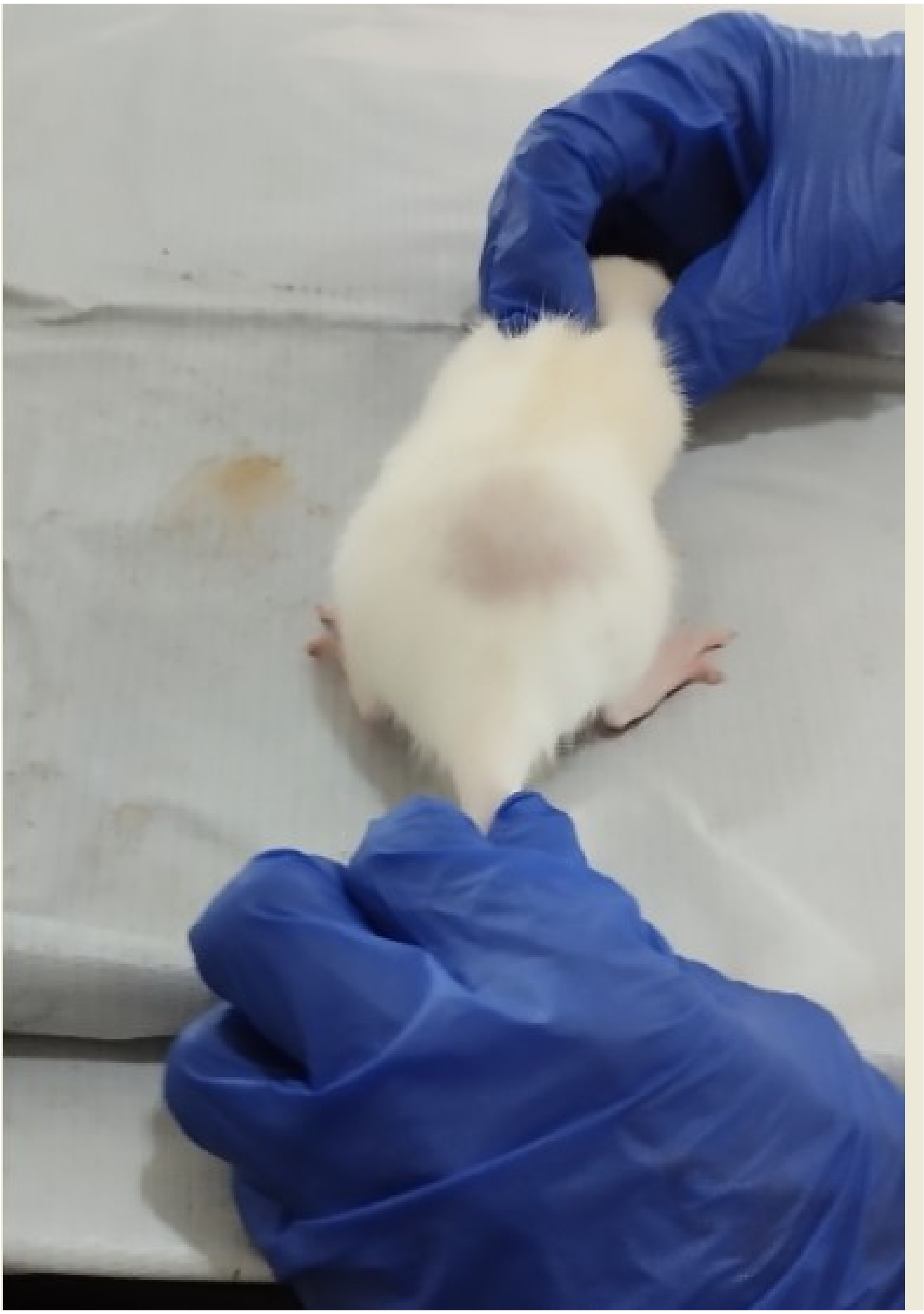

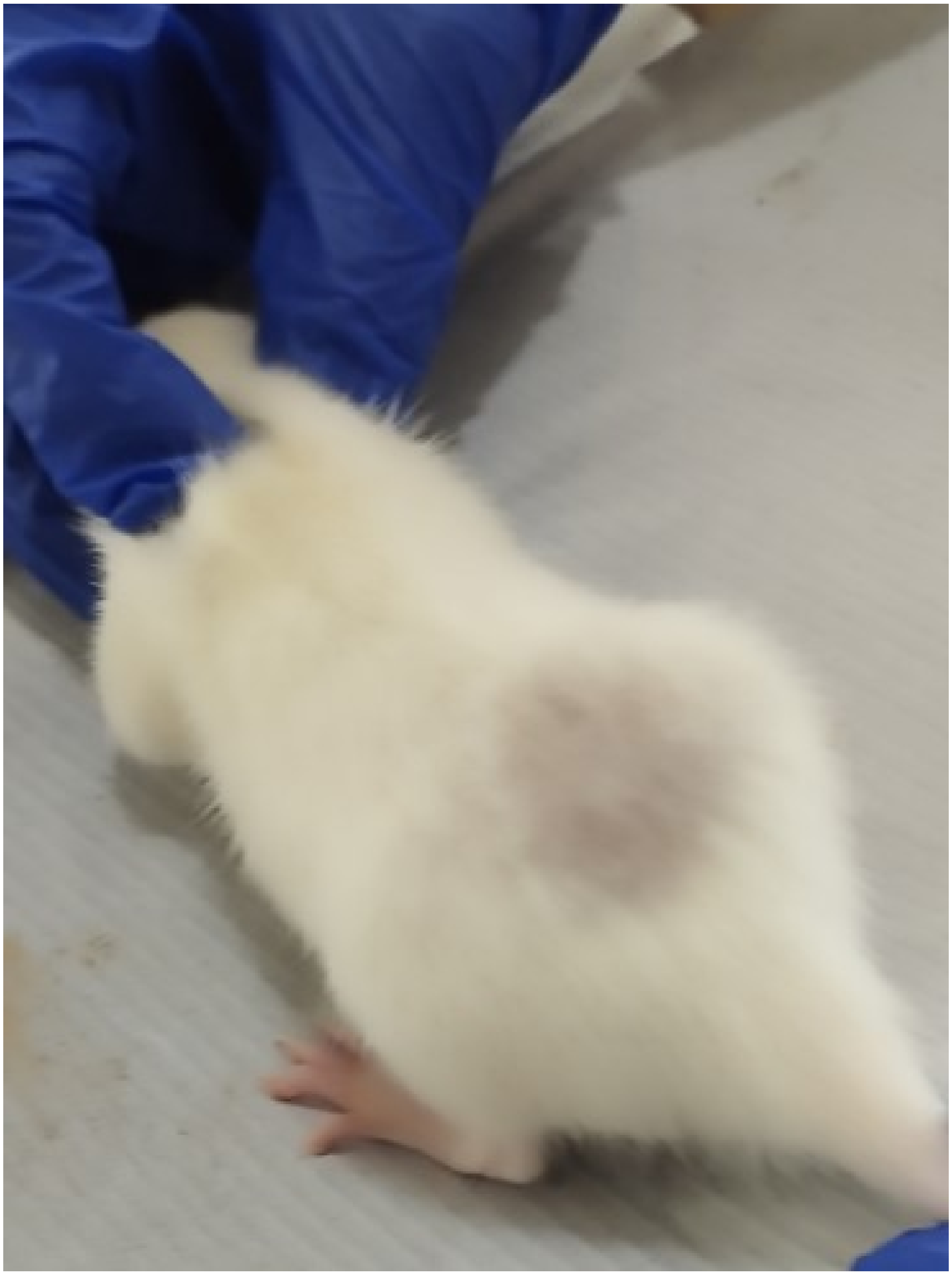

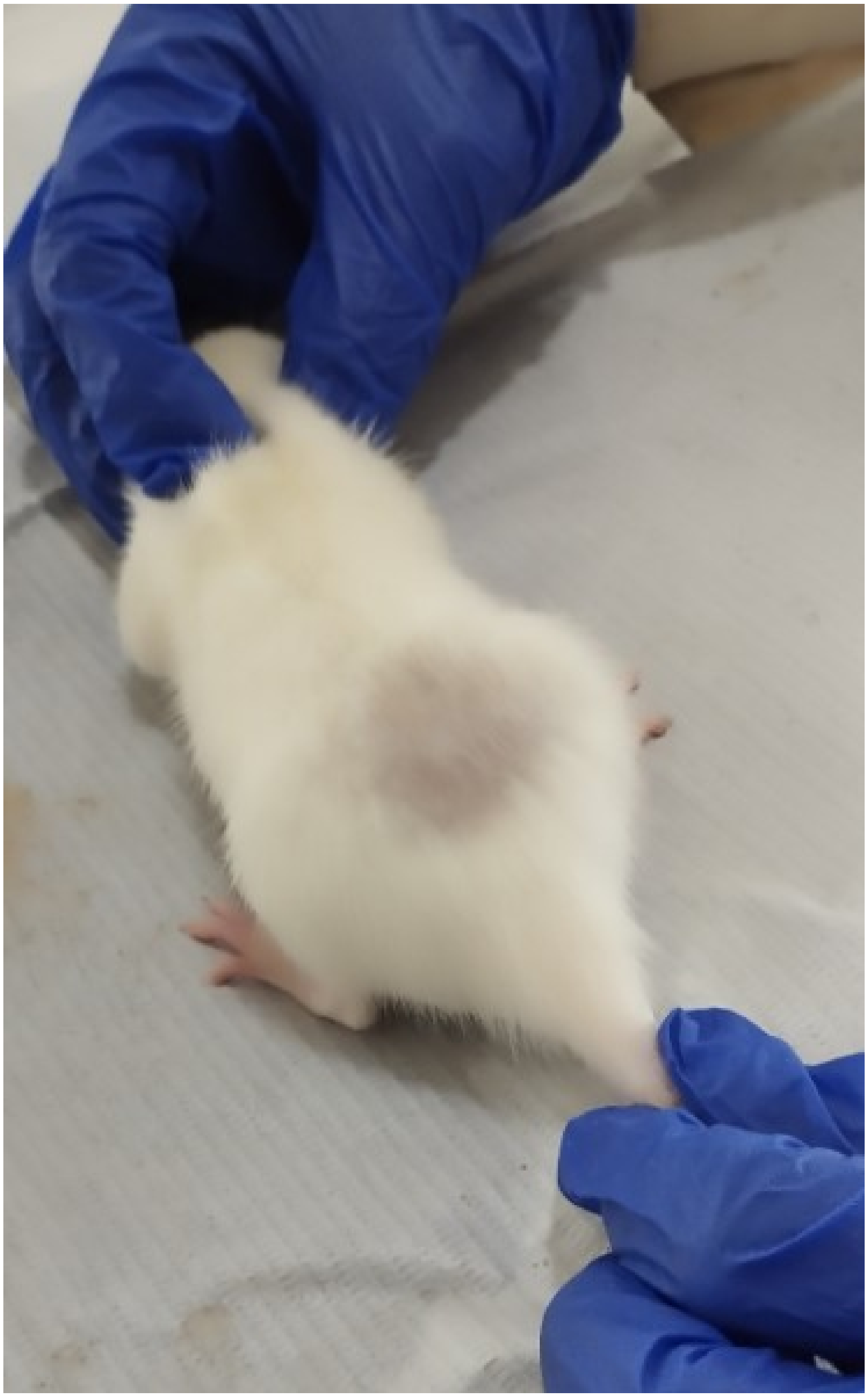

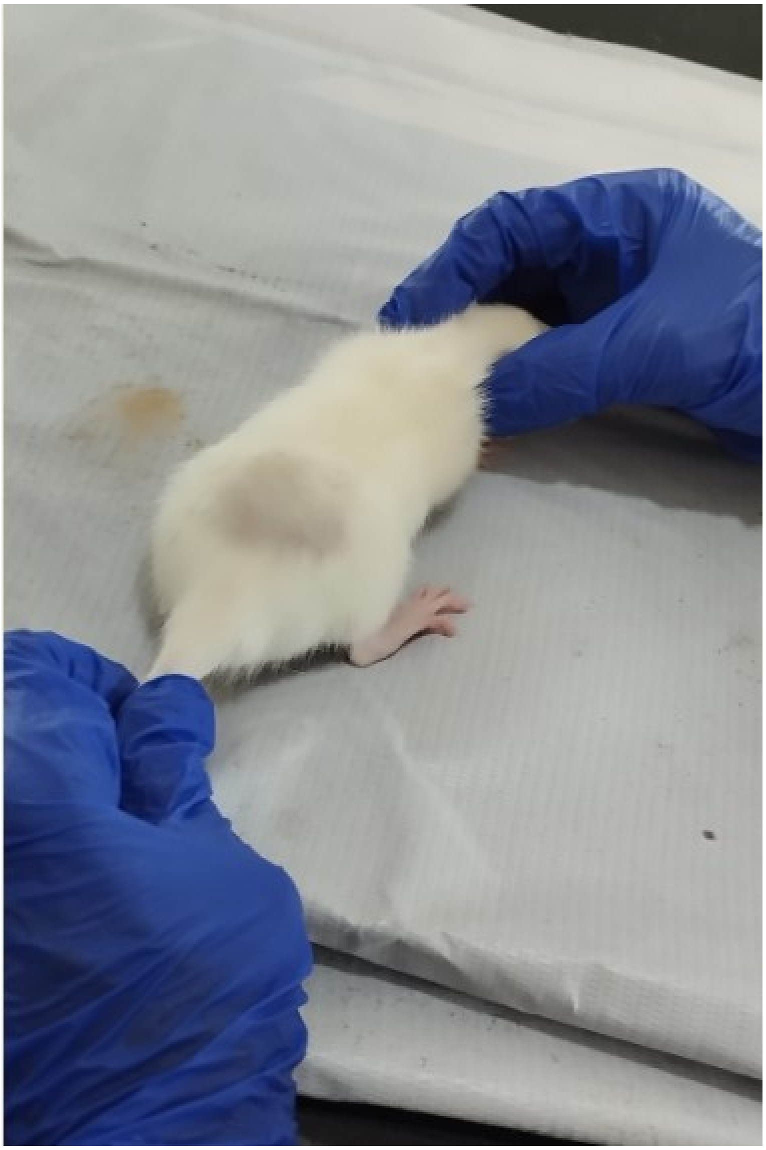

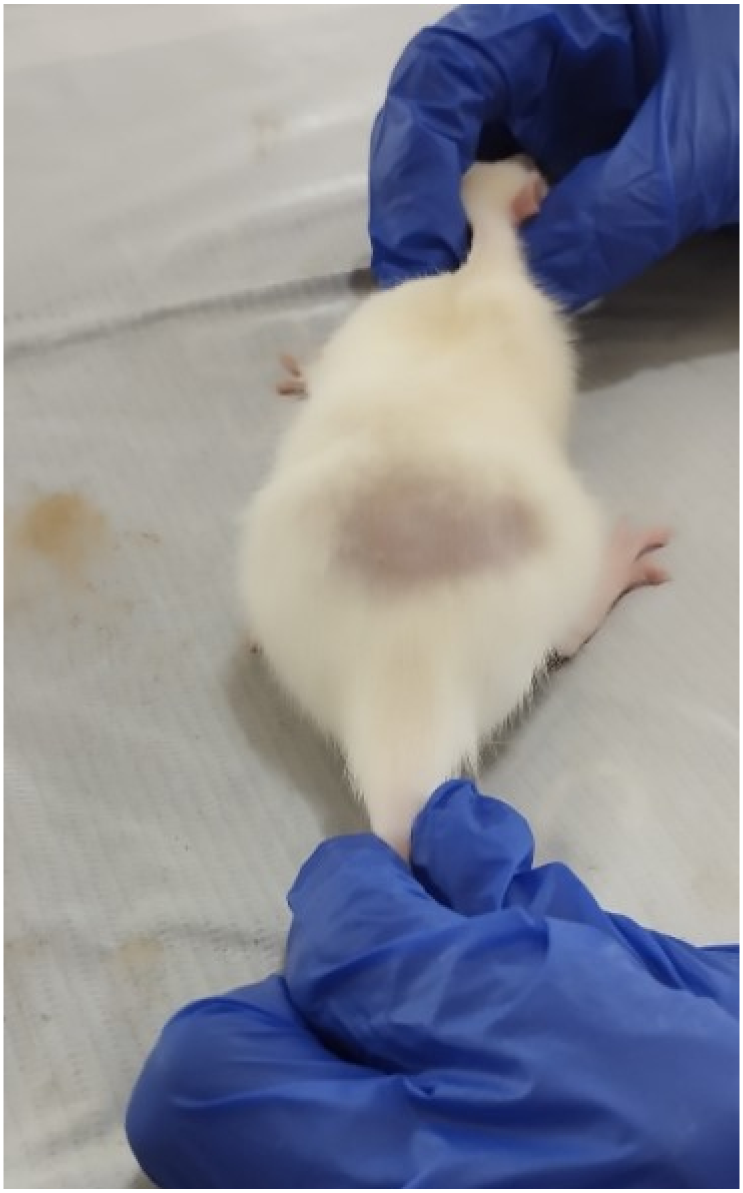

Group II population treated with marketed control

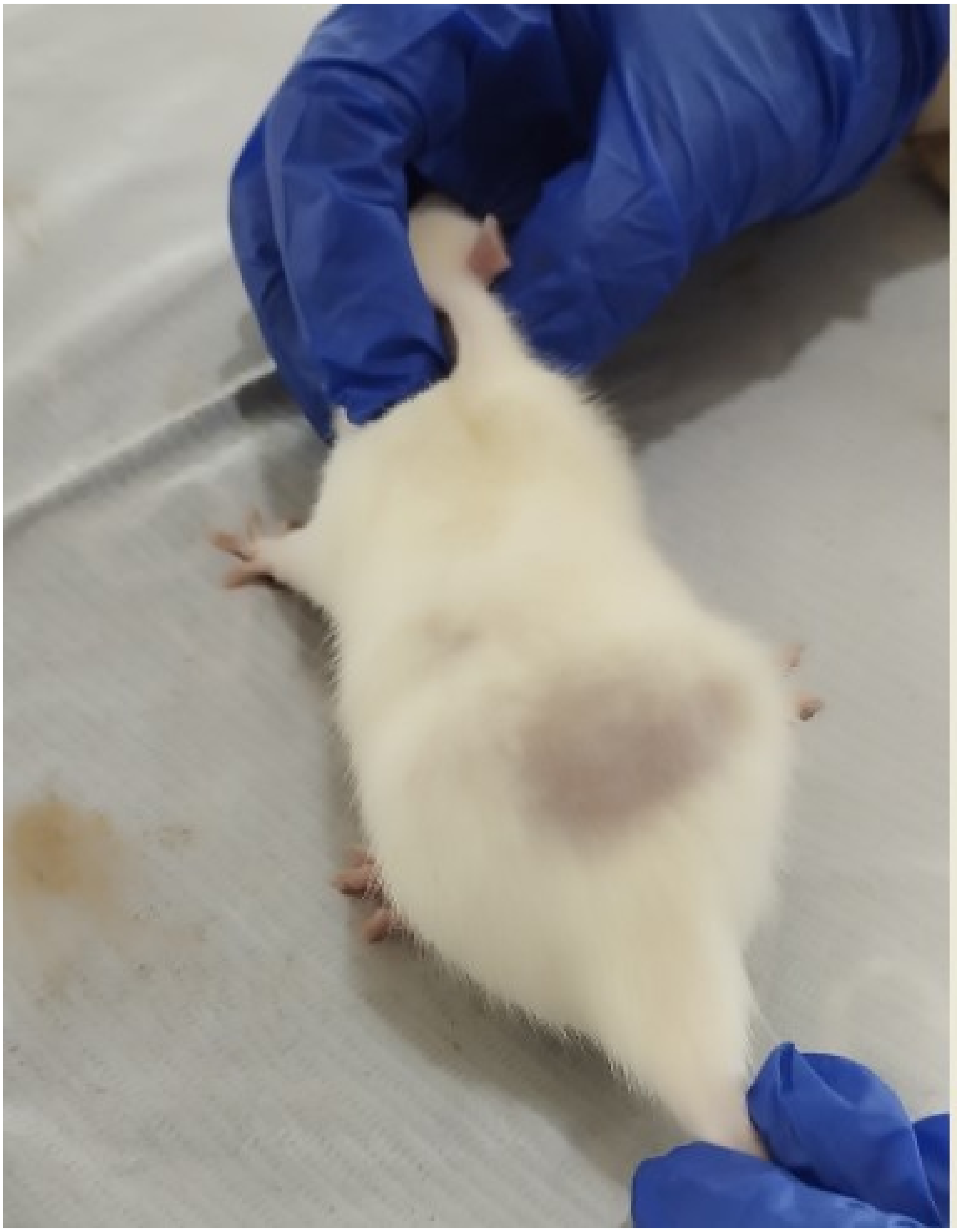

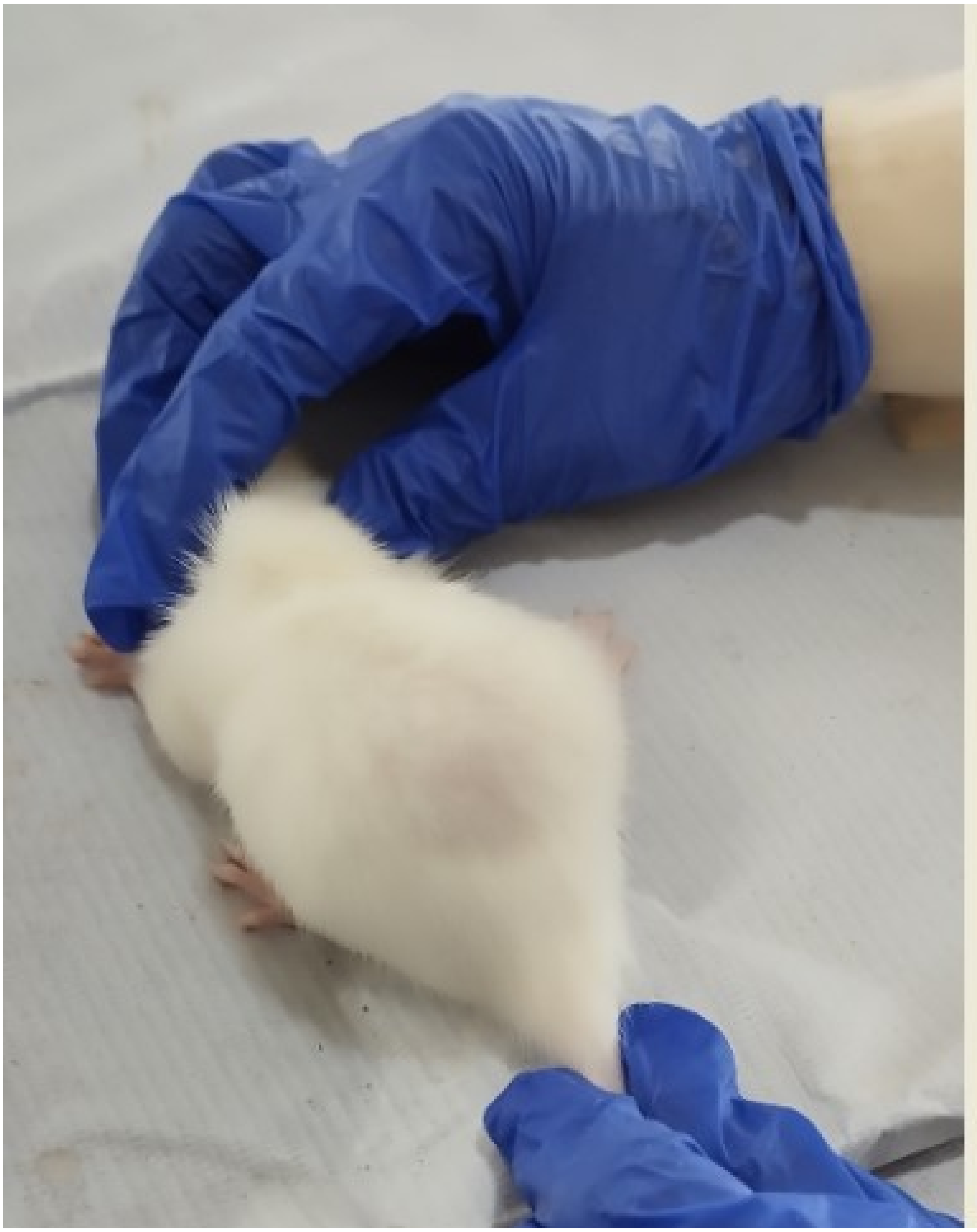

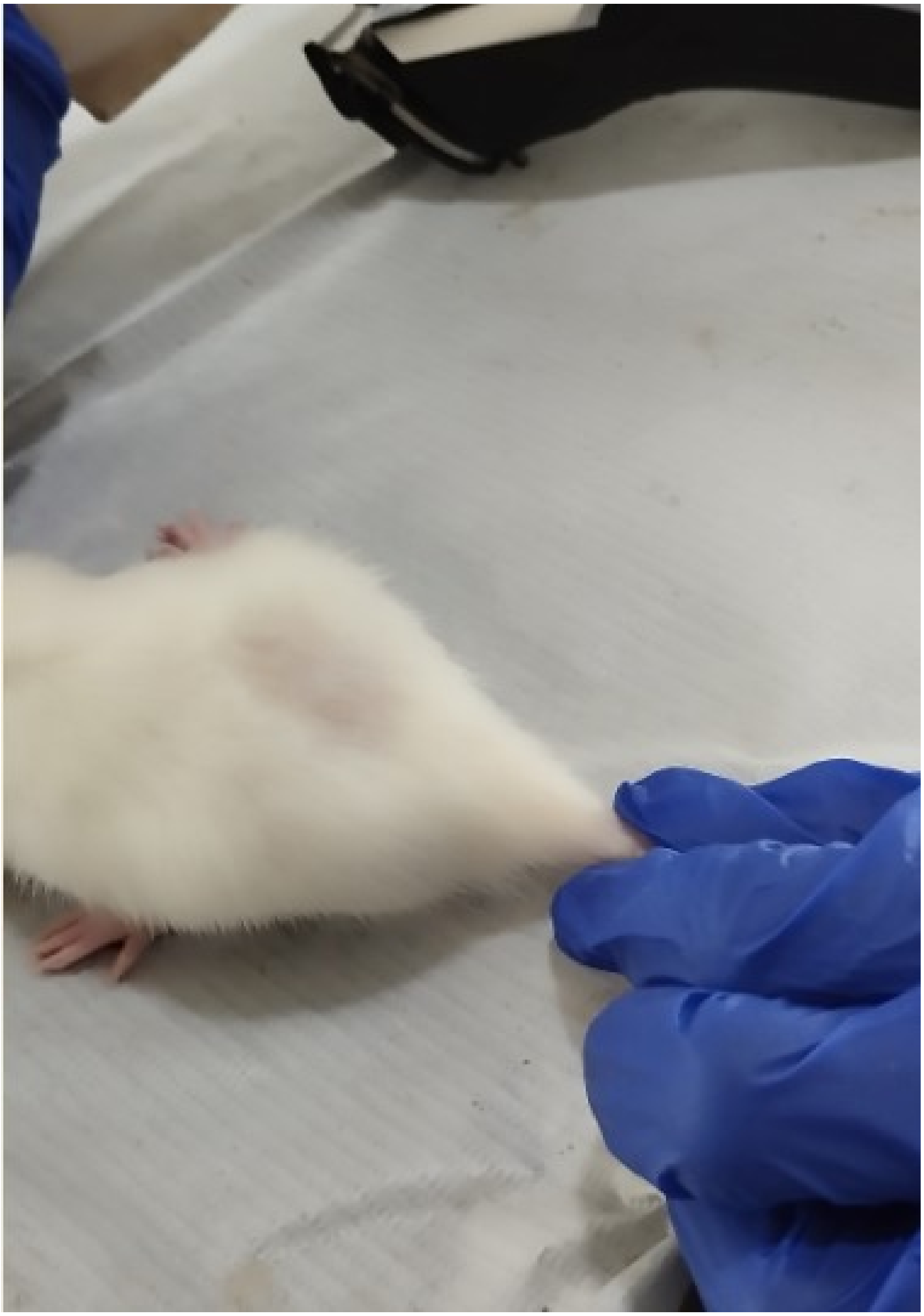

Supplement: RA-015-D5RA00399G-s002 [file RA-015-D5RA00399G-s002.pdf]
